# A Novel FRET Approach Quantifies the Interaction Strength of Peroxisomal Targeting Signals and Their Receptor in Living Cells

**DOI:** 10.3390/cells9112381

**Published:** 2020-10-30

**Authors:** Bernhard Hochreiter, Cheng-Shoong Chong, Andreas Hartig, Sebastian Maurer-Stroh, Johannes Berger, Johannes A. Schmid, Markus Kunze

**Affiliations:** 1 Center for Physiology and Pharmacology, Institute of Vascular Biology and Thrombosis Research, Medical University of Vienna, 1090 Vienna, Austria; bernhard.hochreiter@meduniwien.ac.at; 2 Bioinformatics Institute (BII), Agency for Science, Technology and Research (A*STAR), Singapore 138671, Singapore; chongcs@bii.a-star.edu.sg (C.-S.C.); sebastianms@bii.a-star.edu.sg (S.M.-S.); 3 NUS Graduate School for Integrative Sciences and Engineering, National University of Singapore, Singapore 119077, Singapore; 4 Department of Biochemistry and Cell Biology, Max Perutz Laboratories, University of Vienna, 1030 Vienna, Austria; andreas.hartig@univie.ac.at; 5 Department of Biological Sciences, National University of Singapore, Singapore 117558, Singapore; 6 Center for Brain Research, Department of Pathobiology of the Nervous System, Medical University of Vienna, 1090 Vienna, Austria; johannes.berger@meduniwien.ac.at

**Keywords:** FRET, live-cell measurements, flow cytometry, peroxisomes, PEX5, peroxisomal targeting signal

## Abstract

Measuring Förster–resonance–energy–transfer (FRET) efficiency allows the investigation of protein–protein interactions (PPI), but extracting quantitative measures of affinity necessitates highly advanced technical equipment or isolated proteins. We demonstrate the validity of a recently suggested novel approach to quantitatively analyze FRET-based experiments in living mammalian cells using standard equipment using the interaction between different type-1 peroxisomal targeting signals (PTS1) and their soluble receptor peroxin 5 (PEX5) as a model system. Large data sets were obtained by flow cytometry coupled FRET measurements of cells expressing PTS1-tagged EGFP together with mCherry fused to the PTS1-binding domain of PEX5, and were subjected to a fitting algorithm extracting a quantitative measure of the interaction strength. This measure correlates with results obtained by in vitro techniques and a two-hybrid assay, but is unaffected by the distance between the fluorophores. Moreover, we introduce a live cell competition assay based on this approach, capable of depicting dose- and affinity-dependent modulation of the PPI. Using this system, we demonstrate the relevance of a sequence element next to the core tripeptide in PTS1 motifs for the interaction strength between PTS1 and PEX5, which is supported by a structure-based computational prediction of the binding energy indicating a direct involvement of this sequence in the interaction.

## 1. Introduction

Peroxisomes are ubiquitous, single membrane-bound organelles enclosing a variety of metabolic reactions such as the degradation of various types of fatty acids or hydrogen peroxide (H_2_O_2_). In animals, they are involved in bile acid and ether-phospholipid biosynthesis and in plants in the glyoxylate cycle and photorespiration [1,2]. Accordingly, peroxisomes are essential for most multicellular organisms including humans, where a complete lack of peroxisomal functions causes severe Zellweger spectrum disorders (ZSD), and even single enzyme deficiencies are linked to inherited human diseases [3]. Protein transport of peroxisomal matrix proteins is mediated by two alternative peroxisomal targeting signals (PTS) residing either at the extreme C-terminus (PTS1) [4,5] or close to the N-terminus of the protein (PTS2) [6]. PTS1 motifs are recognized by the soluble receptor protein peroxin 5 (PEX5) [7] and PEX5 transports its cargo proteins across the peroxisomal membrane [8,9]. The PTS1 motif was originally described as the C-terminal tripeptide serine–lysine–leucine (–SKL), but several conservative variants thereof proved functional as well [10,11]. However, a broad variety of C–terminal tripeptides have the potential to bind PEX5 and the amino acid stretch preceding the tripeptide, the upstream sequence, can strengthen or weaken this interaction [12] by exposing the tripeptide due to its unstructured conformation [5,13]. PEX5 consists of a C-terminal tetratricopeptide (TPR) domain binding PTS1-motifs and a long unstructured N-terminal part mediating the transport to the peroxisomal membrane [14,15]. The interaction strength between the TPR–domain and peptides encoding different PTS1 motifs has been amply investigated in vitro [16,17,18,19,20] with a focus on naturally occurring PTS1 motifs and variations within the last three amino acids, whereas the contribution of the upstream sequence has rather been neglected. However, transferring the results of peptide binding assays to the in vivo situation can be problematic, because PTS1 peptides act embedded in full-length cargo proteins and specific properties of the cellular context such as molecular crowding can hardly be mimicked by in vitro systems [21], whereas predictions of this influence are rarely possible [22,23].

The study of protein complexes using purified proteins under artificial in vitro conditions can yield valuable information, but the measurement of PPI within living cells maintains the natural environment of the observed processes. However, such measurements have been limited by the inability to determine the fraction of molecules engaged in binding. Thus, reporter gene-based assays such as two-hybrid (2H) assays often remain the sole access for living cells and serve as valuable tools in spite of their limitations [24,25]. Förster-resonance-energy-transfer (FRET), the radiation-free energy transfer between two fluorophores located in close proximity, can be used to study PPI in living cells [26,27]. The excitation of one molecule (donor) leads to a dipole interaction with the other fluorophore (acceptor) nearby, resulting in emission of photons from the acceptor provided the emission spectrum of the donor overlaps with the absorbance spectrum of the acceptor. The fraction of total energy transferred via FRET, termed FRET efficiency, depends on the distance between the fluorophores and their spatial orientation [28]. However, in living cells the apparent FRET efficiency attributed to a pair of proteins results from the average FRET efficiency of all donor molecules occurring partially in a free and partially in a bound state [29]. Accordingly, the apparent FRET efficiency reflects the saturation state of donor molecules, which depends on the interaction strength of the binding partners, but also the total amount and the molar ratio of donor and acceptor molecules. Disregarding the latter factors causes the broad variability observed in traditional FRET measurements, whereas upon proper normalization the fraction of bound donor molecules is included. Accordingly, in large populations of cells covering a broad spectrum of molar ratios, the apparent FRET efficiency can be plotted as a saturation curve [30]. This allows the implementation of a fitting algorithm based on the law of mass action to extract the underlying parameters of the protein complex.

We have recently introduced a workflow for the 3-filter FRET method [30], in which the intensity of acceptor emission upon donor excitation (FRET-channel) is the critical measure of FRET efficiency. The raw data obtained by this method cannot be used to determine the commonly used physical measure of FRET efficiency (E) directly as the donor, acceptor and FRET intensities exhibit spectral overlaps and are acquired by different measuring modalities (i.e., detector set-ups), which require prior application of compensatory factors. These are obtained by calibrating the 3-filter approach with an equipment insensitive method such as acceptor bleaching using a fusion protein consisting of donor and acceptor, which exhibits a constant FRET and acceptor-to-donor ratio (details on the normalization can be found in Supplementary Text S14.3.5.) [30]. Finally, the value from the FRET-channel is related to the corrected intensity of the donor channel, corrected for its underestimation due to FRET (reduction in donor emission due to energy transfer to the acceptor), to obtain donor-normalized FRET (DFRET) (Supplementary Text S14.3). Thus, DFRET is a 3-filter FRET derived functional equivalent to E, but is used instead of E to account for the indirect nature of its measurement.

These calculations are well-suited for the analysis of data obtained by microscopic approaches, but can develop their full potential in combination with flow cytometry, which generates large data sets and thus improves the power of statistical analyses [31,32,33]. Fitting such data sets according to the law of mass action allows the extraction of fundamental parameters of protein complexes, including the apparent interaction strength (K_a_^app^), but also the stoichiometry factor (z), which describes the average ratio of acceptor and donor within an interaction complex (1 for bimolecular interactions), and the plateau level of the DFRET-saturation curve (FRET_max_), which correlates to the distance between donor and acceptor proteins [30]. As this fitting is performed with normalized intensity values for all three channels, the predicted value for K_a_^app^ is related to the actual association constant K_a_ by a proportionality factor for bimolecular reactions.

In this manuscript, we present the first systematic application of this advanced FRET method for living mammalian cells using the interaction between the TPR domain of human PEX5 and various PTS1 peptides as a model. We demonstrate that our FlowFRET method allows the quantitative determination of an apparent interaction strength measure to estimate relative affinities of PEX5 to different PTS1 peptides. Moreover, we introduce a competition assay using this approach, which can be used for internal confirmation, but also as an independent method resembling biochemical in vitro approaches.

## 2. Materials and Methods

HeLa cells (HeLa-PV, ATCC) and pex5^−/−^ MEF cells [34], kindly provided by M. Baes (Leuven, Belgium), were cultured under standard conditions. FlowFRET experiments and mammalian 2-hybrid assays were performed as described before [30,35] using pex5^−/−^ cells.

FlowFRET was measured on a Cytoflex S flow cytometer (Beckman Coulter). For the 3F-FRET, the donor-channel (excitation^EGFP^→emission^EGFP^) was measured at 488 nm excitation and 525/40 BP (bandpass) emission, the acceptor channel (excitation^mCherry^→emission^mCherry^) at 561 nm excitation and 610/20 BP emission and the FRET channel (excitation^EGFP^→emission^mCherry^) combined 488 nm excitation with 610/20 BP emission. For the competition experiments, the competitor channel (excitation^Cerulean^→emission^Cerulean^) was measured at 405 nm excitation and 450/45 BP emission.

The computational prediction of the interaction strength between PEX5^TPR^ and the different peptides used the FoldX suite based on the PDB structure 1FCH [36].

Further details including plasmid names, cloning procedures, oligonucleotide sequences and detailed experimental descriptions can be found in the supplementary information (Supplementary Text S14).

## 3. Results

### 3.1. A Novel System to Investigate the Interaction between PEX5 and PTS1 by FRET

**Figure 1:**
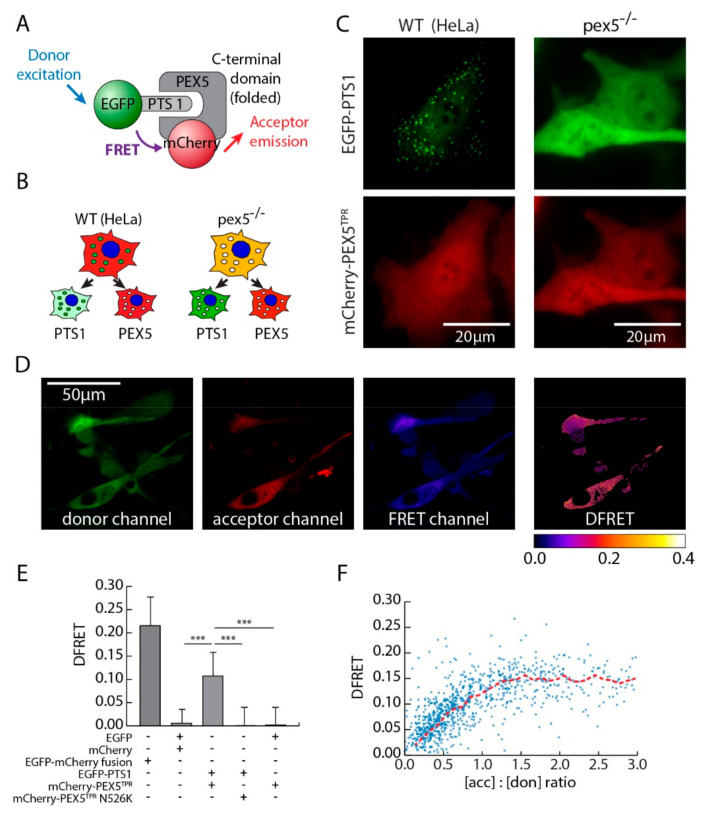
Förster-resonance-energy-transfer (FRET) measurement of the PEX5-PTS1 interaction by fluorescence microscopy. (**A**) Scheme of the interaction and FRET between PTS1-tagged EGFP and the TPR-domain of PEX5 (grey) tagged by mCherry; (**B**,**C**) cytosolic interaction between receptor and targeting signals in the cytosol of cells lacking PEX5: upon co-expression in HeLa cells mCherry-PEX5^TPR^ (red) is cytosolic, whereas EGFP-PTS1 (green) is peroxisomal (left side), but in murine pex5^−/−^ cells both proteins reside in the cytosol (right side); (**D**) microscopy-based 3-filter-FRET experiment using pex5^−/−^ cells transiently expressing mCherry-PEX5^TPR^ and EGFP-PTS1 (Hs55): fluorescence intensity is depicted from left to right in the donor channel, the acceptor channel and the FRET channel(see Materials and Methods for channel definitions), whereas DFRET intensity is depicted by the color-code. (**E**) Quantification of FRET measurements from cells expressing either mCherry-PEX5^TPR^ + EGFP-PTS1 (Hs55) or the positive control (mCherry-EGFP), the negative control (EGFP + mCherry), and one of two controls ablating either the PTS1 binding ability of PEX5^TPR^ (PEX5^TPR^-N526K) or by removing the PTS1 of EGFP (EGFP) using the microscopy based measurement (from left to right: *n* = 648, 129, 537, 190, 150). (**F**) Plotting DFRET values of cells obtained for the interaction between mCherry-PEX5^TPR^ and EGFP-PTS1 against the acceptor to donor ratio ([acc]:[don]) distributes the data similar to a saturation curve (*n* = 537), which is visualized by connecting averages of bins of 0.1 units (red line). Statistics: Kruskal-Wallis test was used with subsequent pairwise testing, *** *p* < 0.001; Ex excitation, Em emission. Description: bars and whiskers in (**E**) depict mean ± sdv.

To study the interaction between PEX5 and diverse PTS1 sequences by FRET measurements, we generated a pair of fusion proteins, one consisting of the PTS1-binding TPR-domain of human PEX5 fused to mCherry (mCherry-PEX5^TPR^) and the other being EGFP extended by a PTS1 motif (EGFP-PTS1) (-GGRRGMGFPAVRSLL, Hs55) [12], which are depicted schematically in Figure 1A. Its unstructured N-terminal part excludes full length PEX5 from being used in such FRET-experiments. The combination of EGFP and mCherry is amply used in cell biological studies, but less common as a FRET pair in spite of proper spectral properties. Under normal conditions, the majority of EGFP-PTS1 is peroxisomal whereas mCherry–PEX5^TPR^ is exclusively cytosolic, because it lacks the critical part of PEX5 mediating peroxisomal transport (Figure 1B,C, left side). However, in a murine cell line devoid of import-competent peroxisomes due to the lack of endogenous PEX5 (pex5^−/−^) [34] EGFP-PTS1 resides in the cytosol as well (Figure 1B,C, right side) and thus its interaction with mCherry-PEX5^TPR^ can be investigated directly. In pex5^−/−^ cells expressing EGFP-PTS1 and mCherry-PEX5^TPR^ both proteins were found evenly distributed across the cytosol, but also an emission of mCherry upon EGFP excitation was observed in the FRET-channel. The FRET efficiency measure *DFRET* was calculated using an mCherry-EGFP fusion protein for normalization (Supplementary Figure S1) as described previously [30], (Figure 1D). Next, the average DFRET value was determined for a large cell population and compared to DFRET values of several controls (Figure 1E). The fusion protein mCherry-EGFP served as positive control and EGFP lacking a PTS1 (EGFP) and a variant of mCherry-PEX5^TPR^ harboring the mutation N526K, which prevents cargo binding [37], were used as negative controls. The average DFRET-value for the interaction between PEX5 and PTS1 was about half of the value of the positive control, whereas in none of the negative controls a signal was detected confirming the specificity of the DFRET measurement in this system. The broad range of DFRET values is caused by the variability in total amount and molar ratio of donor and acceptor proteins among the analyzed cells [30]. When plotting DFRET against the acceptor-to-donor ratio the distribution of data resembles a saturation curve (Figure 1F). Thus, pex5^−/−^-cells are a suitable system to study the interaction between PTS1-carrying proteins and PEX5 by DFRET.

### 3.2. Quantitative Interaction Studies by Flow Cytometry-Based FRET Measurements (FlowFRET)

**Figure 2:**
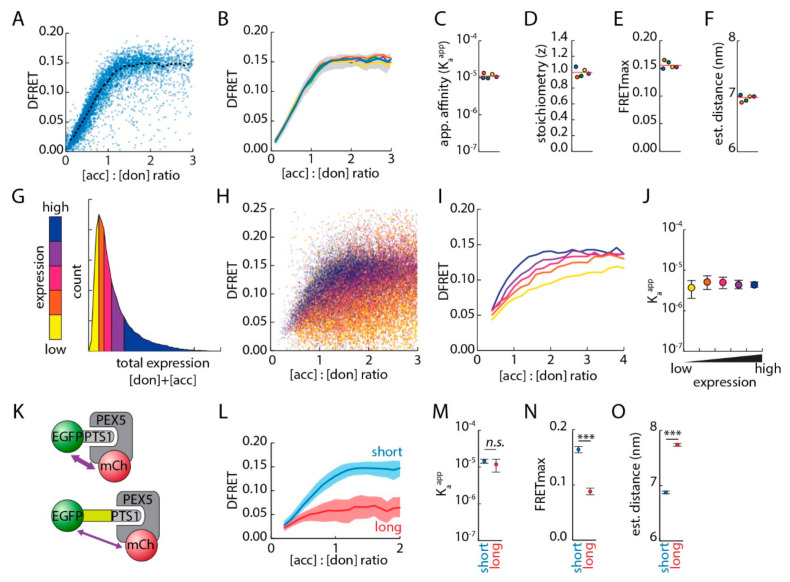
Studying PEX5-PTS1 interaction by high-throughput 3-filter FRET-measurements using a flow cytometer: (**A**) flow cytometer-based fluorescence intensity measurements of pex5^−/−^ cells expressing mCherry-PEX5^TPR^ and EGFP–PTS1 (Hs55) allows the calculation of DFRET values, which are plotted against the acceptor to donor ratio ([acc]:[don]) (*n* = 5000) (blue dots); the saturation curve connects averages of DFRET values for bins of 0.1 units (black dotted line); (**B**) saturation curves of five independent experiments are depicted and the confidence interval of the blue curve is depicted as grey area; (**C**–**E**) fitting the five datasets independently allows the extraction of K_a_^app^, the stoichiometry factor z and the plateau-level of the saturation curve FRET_max_; (**F**) the estimated distance between EGFP and mCherry within the complex is calculated from FRET_max_. (**G**–**J**) The total amount of fluorescent proteins determines the shape of the saturation curve: (**G**) a large dataset was obtained by FlowFRET measurement of pex5^−/−^ cells expressing mCherry-PEX5^TPR^ and EGFP-PTS1 (Hs50), which was subdivided into five equally-sized groups according to the total amount of donor and acceptor proteins; the cells of these groups are depicted by different colors (color-code), and their DFRET values were plotted against the acceptor-to-donor ratio (**H**) or summarized as saturation curves as before (**I**); fitting these subgroups independently resulted in very similar values for K_a_^app^ (mean ± s.e. of the fit) (**J**). (**K**–**O**) Distance between fluorogenic centers is reflected by the plateau level of the DFRET saturation curves: (**K**) schematic representation of an EGFP–PTS1 variant, in which a flexible 10 amino acid linker is inserted in front of the PTS1 (lower picture); (**L**) FlowFRET-measurements of pex5^−/−^ cells expressing mCherry-PEX5^TPR^ together with either EGFP-PTS1 (Hs55) (short, blue) or EGFP-linker-PTS1 (Hs55) (long, red); DFRET saturation curves were obtained by binning the data sets along the *x*-axis and present with different plateau levels. Fitting these data sets results in similar values for the K_a_^app^ (**M**), but clearly different values for FRET_max_ (**N**). (**O**) Predicted spatial distance between EGFP and mCherry. Statistics: (**C**–**F**) (from left to right: *n* = 13,484, 12,728, 12,642, 25,848, 19,239) Description: error bars in (**J**) depict standard error (s.e.) of the fitting; (**M**–**O**): unpaired t-test, *n* = 5; *** *p* < 0.001, *n.s.* not significant.

Extracting quantitative information about this protein complex is achieved by a fitting algorithm based on the law of mass action, which uses FRET-corrected intensity values for donor- and acceptor proteins together with DFRET values reflecting the fraction of acceptor-bound donor proteins, but requires large data sets for high statistical power. As a combination of flow cytometry and FRET efficiency measurements is highly suitable to provide such data sets [30], we used a cytometer with appropriate excitation lasers and detection systems (Supplementary Figure S2 and Supplementary Text S14.3) to attribute a set of individual intensities in donor, acceptor and FRET channel to a large number of cells. When we measured a mixture of pex5^−/−^ cell pools, each transfected with a different ratio of expression plasmids for mCherry-PEX5^TPR^ and EGFP-PTS1, the DFRET values displayed as a saturation curve when plotted against the ratio of acceptor and donor concentrations (corrected for the loss of donor intensity due to FRET) (Figure 2A and Supplementary Text S14.3.5.).

For better visualization, individual values were also aggregated into bins along the *x*-axis and independent repetitions of this experiment resulted in very similar curves demonstrating the high reproducibility of this method (Figure 2B and Supplementary Figure S3). Next, these data sets were independently subjected to the fitting algorithm to extract K_a_^app^ (Figure 2C), z (Figure 2D), and FRET_max_ (Figure 2E) for each of the data sets (Supplementary Text S14.3.7.) [30]. The predicted values were very similar as was the predicted distance between donor and acceptor molecules in the complex (Figure 2F), which can be recalculated from FRET_max_ values based on the relation between distance and transfer efficiency in FRET experiments (Supplementary Text S14.3.8.) [38]. It is important to note that distances measured via FRET, especially for large fluorescent proteins, represent rough estimates and should only be viewed as relative measures. K_a_^app^ is a correlative measure for the interaction strength and is directly related to the K_a_, but cannot be simply recalculated unless the proportionality factor relating the measured intensity and the real protein concentration is known (for detail, [30]). The whole procedure as specified above is termed FlowFRET from here on. Large data sets were also required to confirm predictions of our mathematical model, which suggests that DFRET values change with the total amount of donor and acceptor protein, thus affecting the progression of saturation curves [30]. We performed a similar FlowFRET experiment using another EGFP-PTS1 variant (-IAMNCVQCKSQL, Hs50, [12]) with lower affinity for PEX5, which should increase the differences in curve progression upon changes in total protein level. The data set was subdivided into five groups according to the combined intensity of donor and acceptor molecules (Figure 2G, Supplementary Figure S4). The different groups were well separable when the DFRET values were plotted against the acceptor-to-donor ratio (Figure 2H), but also when the data set for each group was depicted as independent saturation curves (Figure 2I). Nonetheless, when these data sets were fitted independently, the predicted K_a_^app^s were very similar (Figure 2J). This confirmed that DFRET values depend on the total amount of interacting proteins, but also demonstrated the power of the fitting approach.

As FRET efficiency depends on the *distance* between donor and acceptor proteins, the FRET_max_ levels of DFRET saturation curves should be lower when the distance between donor and acceptor molecules is artificially increased. To verify this prediction, an additional peptide sequence (Y-G-G-G-S-G-G-S-G-G), composed of amino acids characteristic for unstructured linker domains, was inserted between the PTS1 and EGFP of the donor protein to obtain EGFP-linker-PTS1 (Hs55) (Figure 2K). FlowFRET experiments were performed as before using pex5^−/−^ cells expressing mCherry-PEX5^TPR^ together with either EGFP-PTS1 (short) or EGFP-linker-PTS1 (long). As expected, the resulting saturation curves differed markedly (Figure 2L, Supplementary Figure S5), as the plateau levels of DFRET were drastically lower in the presence of the linker. Fitting five independent data sets, we found that the predicted K_a_^app^ values remained similar (Figure 2M), but the predicted FRET_max_ values were significantly lower upon insertion of the peptide (Figure 2N), which reflects an increase in the predicted distance between donor and acceptor molecules (Figure 2O). The numerical increase of about 1 nm (10 A) is in line with the expectation, based on a calculated increase of about 3.6nm for a peptide of 10 amino acids in elongated conformation, which is reduced to an average length of about 30% by the flexibility of the backbone in an unstructured peptide [39]. These results demonstrate the ability to identify changes in the distance and the insensitivity of K_a_^app^ predictions to changes in the FRET_max_ value.

### 3.3. Discrimination of Affinities by FlowFRET 

**Figure 3:**
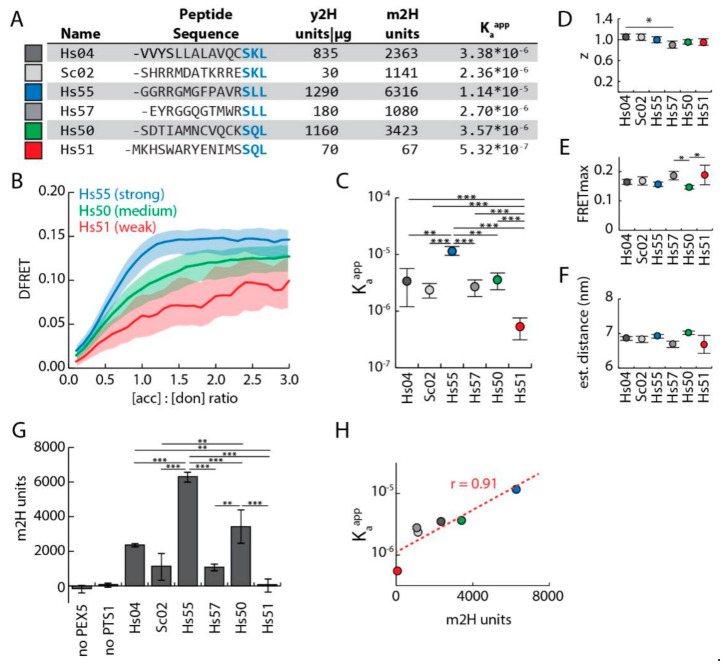
Affinities of PEX5 for different PTS1 variants can be discriminated by FlowFRET: (**A**) Table of six different PTS1 peptides investigated, together with the apparent interaction strength measured by a yeast 2H assay (y2H [12]), by the mammalian 2H assay (m2H, cf. Figure 3F) and by FlowFRET (K_a_^app^); (**B**) different curve progression for strong, intermediate and weak PTS1: FlowFRET measurements of pex5^−/−^ cells expressing mCherry-PEX5^TPR^ together with one of the six EGFP-PTS1 variants are represented by saturation curves for PTS1 peptides with a high (Hs55, blue), intermediate (Hs50, green) or low (H51, red) affinity to PEX5^TPR^; (**C**–**E**) independent fittings of data sets resulted in markedly different predictions for the K_a_^app^, but very similar ones for the stoichiometry factor (z) and the plateau level of the saturation curve (FRET_max_) (*n* = 3, mean ± standard deviation (sdv)). (**F**) The estimated distance between mCherry and the different EGFP proteins was calculated from FRET_max_ values. (**G**) m2H: normalized luciferase activity of pex5^−/−^ cells expressing PEX5^TPR^ behind the activation domain of the Herpes-Simplex-Virus protein-16 (VP16^AD^-PEX5^TPR^) and EGFP-PTS1 variants fused to the DNA-binding domain of the yeast Gal4p protein (GAL4^DBD^-EGFP-PTS1) each terminating with the peptide described above or controls expressing VP16^AD^- or GAL4^DBD^- fusion proteins alone is presented (*n* = 3, mean ± sdv); (**H**) correlation of the results of FlowFRET (K_a_^app^) and the apparent interaction strength obtained by the m2H in pex5^-/-^ cells. Statistics: One-way ANOVA for log10 of K_a_^app^ (**C**), z (**D**) and FRET_max_ (**E**), ((**C**–**F**): three independent experiments’ mean ± sdv). *** *p* < 0.001, ** *p* < 0.01, * *p* < 0.05, Description: (**B**) Lines represent moving average, shaded area represents the upper and lower quartiles.

To verify that our novel approach is able to discriminate different affinities, we extended our sample by four additional PTS1 peptides, presenting with drastically different affinities to PEX5 in a semi-quantitative yeast-2H or a peptide binding assay [12] (Figure 3A). pex5^−/−^ cells expressing mCherry-PEX5^TPR^ and one of the six EGFP–PTS1 variants were analyzed by FlowFRET (Supplementary Figure S6). The shape of three representative saturation curves for a strong (Hs55), a medium (Hs50) and a weak (Hs51) PEX5-binding peptide are clearly different (Figure 3B), especially in the ascending phase at low acceptor-to-donor ratios. Although some of the curves never reached the plateau, fitting of all data sets resulted in significantly different K_a_^app^ values reflecting different affinities for the peptides (Figure 3C, numerical values cf. Figure 3A). In contrast, the values for z and FRET_max_ were highly similar and the predicted distances were nearly indistinguishable (Figure 3D–F). Differences in FRET_max_ might partially result from altered flexibility of the upstream domain affecting the actual distance between the fluorogenic centers. To compare our approach with an independent method in the same cells, we performed a mammalian 2H assay (m2H). To that end, we generated expression plasmids for PEX5^TRP^ fused to the herpes simplex VP16-activation domain (*prey*, VP16^AD^-PEX5^TPR^) and for the various EGFP-PTS1 variants fused to the Gal4p DNA-binding domain (*bait*, pM-EGFP-PTS1) and co-expressed them with a luciferase and a β-galactosidase reporter plasmid in pex5^−/−^ cells. When normalized luciferase activity (luciferase/β–galactosidase) was determined for the cell extracts, we obtained clear differences for the EGFP-PTS1 variants, but no activity was obtained when only one binding partner was expressed (Figure 3G). The K_a_^app^ values obtained by FlowFRET and the m2H units correlated well (Figure 3H), whereas the correlation of K_a_^app^ values with the units obtained by a yeast-2H assay using peptides alone was less strong (Supplementary Figure S7). 

### 3.4. Live-Cell Competition Experiments by FlowFRET 

**Figure 4:**
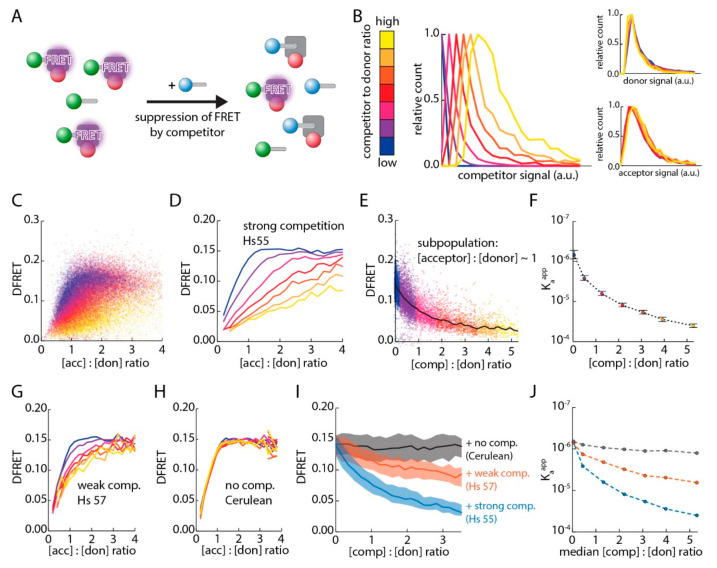
Establishing a live-cell competition assay: (**A**) Schematic representation of a FRET-based competition assay: Cerulean-PTS1 (blue) reduces the fraction of mCherry-PEX5^TPR^ (red) engaged in a FRET-producing interaction with EGFP-PTS1 (green); (**B**–**F**) FlowFRET measurements of pex5^−/−^ cells expressing mCherry-PEX5^TPR^ and EGFP-PTS1 (Hs55) together with varying amounts of Cerulean-PTS1 (Hs55); seven equally sized populations of cells were generated according to the competitor to donor ratios and thus the average amount of competitor protein was clearly different among these populations (left panel), whereas the distribution of donor and acceptor were comparable (right panels) (**B**); subpopulations were highlighted by color-code and are depicted by the DFRET values of individual cells (**C**) or by the saturation curves (**D**); (**E**) when plotting the DFRET values of cells sharing acceptor-to-donor ratios of around one (0.8 < x < 1.2) against the competitor to donor ratio, a traditional decay curve is obtained; (**F**) fitting the seven data sets independently (cf. (**B**)) allows the extraction of their K_a_^app^s, which were plotted against the competitor-to-donor ratio (fitting estimate ± s.e. of fit); (**G**,**H**) saturation curves of corresponding competition experiments utilizing a competitor with lower affinity to PEX5 (Cerulean-PTS1, Hs57) (**G**) or Cerulean alone (**H**); (**I**) DFRET decay curves for competitors with high (Hs55, blue) or low affinity (Hs57, red) to PEX5^TPR^ or for Cerulean alone (grey) using cells with an acceptor-to-donor ratio of around one; (**J**) fitting equally sized data sets allows the extraction of K_a_^app^ in the presence of different competitor-to-donor ratios for Cerulean–PTS1 with a high (blue) or low (orange) affinity to PEX5 or for Cerulean alone (grey). Statistics: (**B**–**F**) (*n* = 39,698).

Next, we wanted to establish an in vivo equivalent to competition experiments, which have been amply applied in in vitro approaches. However, our analysis involves cells with varying concentrations of donor, acceptor and competitor proteins. Thus, an estimation of the effectivity of competition not only has to consider the molar ratio of competitor and donor, but also the expected FRET efficiency based on the abundance of donor and acceptor proteins. To determine the amount of the competitor for each cell, we took advantage of Cerulean, a third type of fluorescent protein with excitation and emission properties at short wavelengths [40]. Using a fusion protein of mCherry and Cerulean (mCherry-Cerulean), we extracted a normalization factor to calculate the molar ratio of acceptor and competitor molecules from the intensities in the respective channels. The calculation of this normalization factor also accounts for the FRET that occurs between competitor and acceptor in the fusion protein (Supplementary Text S14.3.6.). First, we investigated whether the ectopic expression of Cerulean harboring the same PTS1 (Hs55) affects K_a_^app^ obtained for EGFP-PTS1 (Hs55) and mCherry-PEX5^TPR^. For that purpose, we performed FlowFRET experiments for pex5^−/−^ cells expressing mCherry-PEX5^TPR^ and EGFP-PTS1 together with Cerulean-PTS1 (Hs55 (Figure 4A)). The intensity in the Cerulean channel (see Materials and Methods for channel definition) was independently determined for each cell to calculate the molar ratio of donor and competitor via the normalization factor. Next, we subdivided this data set into seven groups according to the competitor to donor ratios and confirmed that these populations demonstrate clearly different average levels of competitor, but very similar distributions in the levels of donor and acceptor molecules (Figure 4B). When plotting DFRET results for the whole data set against the acceptor-to-donor ratio, it became evident that the relative abundance of competitor, as defined by the different subgroups, affected the shape of the data clouds (Figure 4C). Moreover, the progressions of the saturation curves were clearly different (Figure 4D), suggesting different effective K_a_^app^s due to the competing effect of Cerulean-PTS1. To depict the decay of DFRET upon different competitor-to-donor ratios, we selected a subset of data with an acceptor-to-donor ratio of around one (equimolar donor and acceptor) and plotted DFRET against the competitor-to-donor ratios as described before (Figure 4E). 

The shape of the resulting curve clearly resembles the logarithmic decay of a traditional inhibitory curve. Next, the data sets of the subgroups were fitted independently and their K_a_^app^s were plotted against the competitor to donor ratio resulting in a similar decay curve (Figure 4F). Finally, we compared the effects of competitors with different binding strengths by expressing a Cerulean-PTS1 variant with a drastically lower affinity to PEX5 (Hs57, cf. Figure 3) or Cerulean alone. Similar FlowFRET competition experiments were performed and the saturation curves reflecting different competitor-to-donor levels obtained for Cerulean-PTS1 (Hs57) were clearly different from each other (Figure 4G, Supplementary Figure S8), but more similar than the curves observed with the high affinity competitor Cerulean-PTS1 (Hs55). In contrast, when Cerulean alone was used as competitor, the saturation curves were indistinguishable (Figure 4H, Supplementary Figure S8E–H). Using subgroups with a donor-to-acceptor level around one, we found that the decay of DFRET values with increasing competitor-to-donor ratio was steepest for the high affinity competitor (Hs55), less steep for the low affinity competitor (Hs57), and Cerulean alone did not change DFRET across the whole range of competitor to donor ratios (Figure 4I). Correspondingly, the predicted K_a_^app^ values of independently fitted subpopulations were reduced most effectively by the high affinity competitor (Hs55), and less effectively by the weak affinity competitor (Hs57), but were not reduced by Cerulean alone (Figure 4J). 

### 3.5. The Upstream Sequence of PTS1 Motifs Determines Their Binding Strength to PEX5

**Figure 5:**
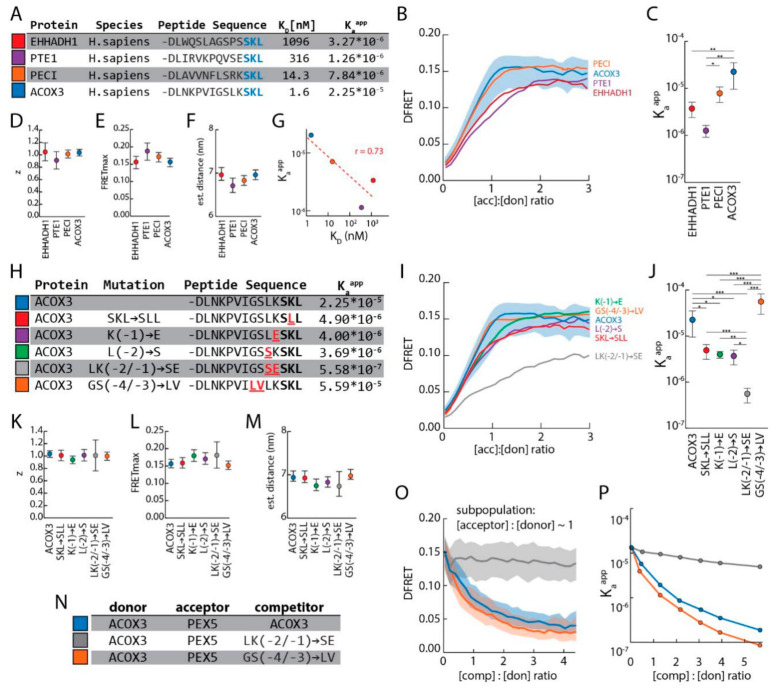
The upstream sequence of PTS1 motifs strongly modulates the interaction strength with PEX5: (**A**) Peptide sequences encoding PTS1 motifs of human proteins terminating with -SKL; the affinity of the peptides obtained by in vitro binding experiments [16], and K_a_^app^s obtained by FlowFRET (described below) are indicated; (**B**–**F**) FlowFRET measurements of pex5^−/−^ cells expressing mCherry-PEX5^TPR^ and one of the EGFP-PTS1 variants; saturation curves are depicted together with the quartiles for the PTS1 of ACOX3 (**B**); fitting the data sets allowed the extraction of K_a_^app^ (**C**), z (**D**) and FRET_max_ (**E**), from which the distance between mCherry and EGFP was predicted (**F**); (**G**) correlation between the apparent interaction strength obtained by in vitro peptide binding assays (depicted as K_D_) or by FlowFRET (depicted as K_a_^app^); (**H**) peptide sequences encoding the PTS1 of human ACOX3 and variants thereof; K_a_^app^s obtained by FlowFRET (below); (**I**–**M**) FlowFRET experiment studying the interaction between PEX5^TPR^ and ACOX3 mutants; (**I**) saturation curves were obtained as before and quartiles for the PTS1 of ACOX3 are indicated; fitting the data sets allowed the extraction of K_a_^app^ (**J**), z (**K**) and FRET_max_ (**L**), and the distance between mCherry and EGFP in the complex was calculated (**M**). (**N–O**) Live-cell competition to investigate the interaction between PEX5^TPR^ and three PTS1 of ACOX3 variants: FlowFRET experiments of pex5^−/−^ cells expressing mCherry-PEX5^TPR^ and EGFP-PTS1 (ACOX3) together with one of the Cerulean-PTS1 variants (ACOX3 (blue), ACOX3-^-4^GS^-3^/LV (grey) or ACOX3-^-2^LK^-1^/SE (orange) (**N**)) were performed as described before; (**O**) decay of DFRET values with increasing competitor-to-donor ratios using cells with an acceptor-to-donor ratio of around one (0.8 < x < 1.2) and (**P**) reduction in K_a_^app^, when different populations of competitor-to-donor ratios are fitted independently. Statistics: One-way ANOVA for log_10_ of K_a_^app^ (**C**), z (**D**) and FRET_max_ (**E**), ((**C**–**F**): three independent experiments, mean ± sdv); one-way ANOVA for log_10_ of K_a_^app^ (**J**), z (**K**) and FRET_max_ (**L**), ((**I**–**M**): *n* = 4); (**N**–**O**): *n* = 105,306 (ACOX3), 105,135 (LK/SE) and 127,442 (GS/LV); *** *p* < 0.001, ** *p* < 0.01, * *p* < 0.05, Description: (**B**,**I**) Lines represent binned average, shaded area represents the upper and lower quartiles.

Next, we used FlowFRET to investigate the contribution of the upstream sequence to the quality of PTS1 motifs, because our approach properly reflects the cytosolic environment of the interaction and the proteinaceous context of PTS1 peptides. The latter might be important to evaluate the functional relevance of structural flexibility in the upstream region fostering the exposition of the C-terminal tripeptide. We selected four PTS1 sequences occurring in human peroxisomal proteins, which share the C-terminal tripeptide (-SKL), but differ markedly in the preceding nine amino acids (acyl-CoA oxidase 3 (ACOX3), peroxisomal enoyl-CoA isomerase (PECI), peroxisomal thioesterase 1 (PTE-1), and L-bifunctional enzyme (EHHADH)), (Figure 5A). In an in vitro peptide-binding assay, these peptides bind with very different affinities (K_d_s for PEX5^TPR^ are 1.6 nM, 14.3 nM, 316 nM and 1096 nM, respectively) to the TPR domain of PEX5 [16] although they all were sculptured by evolutionary adaptations. In FlowFRET experiments using EGFP-PTS1 variants, each terminating in one of these four peptides, the ascending part of the saturation curves displayed marked differences in the slope, but converged at a similar plateau level (Figure 5B and Supplementary Figure S9A,B).

Accordingly, the predicted K_a_^app^ values were clearly different (Figure 5C), whereas the values for z and FRET_max_ were very similar as was the predicted distance between the fluorogenic centers (Figure 5D–F). Overall, the predicted K_a_^app^ values correlated with the in vitro affinities obtained for the corresponding peptides and PEX5^TPR^ [16] (Figure 5G), except for K_a_^app^ of EHHADH, which had a markedly higher affinity in our measurements. Next, we retraced the contribution of the upstream sequence to individual positions studying the effects of individual point mutations in the PTS1 of ACOX3 (Figure 5H). All mutations were expected to deteriorate the quality of the PTS1 based on the in silico evaluation of putative PTS1 motifs (*PTS1–predictor* [41]). Two mutations are in close proximity to the C-terminal tripeptide, ^-1^K/E, ^-2^L/S, and their combination (^-2^LK^-1^/SE) allows testing for additive effects. Two other mutations are located further upstream and substitute the flexible residues glycine and serine by the hydrophobic residues leucine and valine (^-4^GS^-3^/LV). As additional control, we substituted the typically positive residue at the center of the tripeptide (-SKL) with a hydrophobic one (-SLL), which apparently contradicts the observable preference in naturally occurring PTS1, but similar tripeptides had been identified in peptides with high affinity to PEX5 using a yeast-2H assay [12]. We performed FlowFRET measurements using mCherry-PEX5^TPR^ and the different EGFP-PTS1 variants harboring either the native PTS1 of ACOX3 or mutated forms thereof. The saturation curves displayed different curve progressions in the ascending phase, but converged at a similar plateau level except for the variant ^-2^LK^-1^/SE (Figure 5I and Supplementary Figure S9C,D). The predicted K_a_^app^ values of nearly all ACOX3 mutations were markedly lower than that of the original ACOX3-PTS1 with the double mutant ^-2^LK^-1^/SE drastically reducing K_a_^app^ for PEX5^TPR^ (Figure 5J). Only the variant ^-4^GS^-3^/LV presented with an increased K_a_^app^, although it was predicted to drastically reduce the quality of the PTS1. In contrast, the predicted values for z and FRET_max_ were highly similar as was the predicted distance between EGFP and mCherry (Figure 5K–M). Thus, even single mutations in the upstream sequence can drastically change the interaction strength between PEX5 and a cargo protein and the atypical tripeptide (-SLL) still mediates the interaction in living cells. To corroborate the surprising effect of the double mutation ^-4^GS^-3^/LV, we performed a FlowFRET-based competition experiment comparing the ability of critical ACOX3 variants to act as competitor. Thus, we co-expressed mCherry-PEX5^TPR^ and EGFP-PTS1 (ACOX3) together with Cerulean variants terminating either in the native PTS1 of ACOX3, the high affinity variant ^-4^GS^-3^/LV (ACOX3^GS/LV^) or the low affinity variant ^-2^LK^-1^/SE (ACOX3^LK/SE^) (Figure 5N and Supplementary Figure S10). From the data sets obtained by FlowFRET measurements (Supplemenrary Figure S11), we selected subsets of cells with an acceptor-to-donor ratio of around one and plotted the decay curve of DFRET values against the competitor-to-donor ratio (Figure 5O and Supplementary Figure S10C,H,M). ACOX3 variants with high affinity for PEX5 (ACOX3 and ACOX3^GS/LV^) displayed steep decay curves, whereas the weak PTS1 (ACOX3^LK/SE^) hardly caused a reduction in DFRET. Next, each of the data sets was subdivided into seven groups according to the competitor to donor ratio and the K_a_^app^s of each group was predicted independently by fitting. The K_a_^app^s declined rapidly, when increasing amounts of Cerulean harboring the PTS1 of either ACOX3 or ACOX3^GS/LV^ were expressed, but hardly changed when the competitor harbors the PTS1 of ACOX3^LK/SE^ (Figure 5P and Supplementary Figure S10E,J,O). As the slope of the decay curve for ACOX3^GS/LV^ is even steeper than that for ACOX3, its K_a_^app^ is higher, supporting the results of the direct measurements.

### 3.6. Computational Verification 

**Figure 6:**
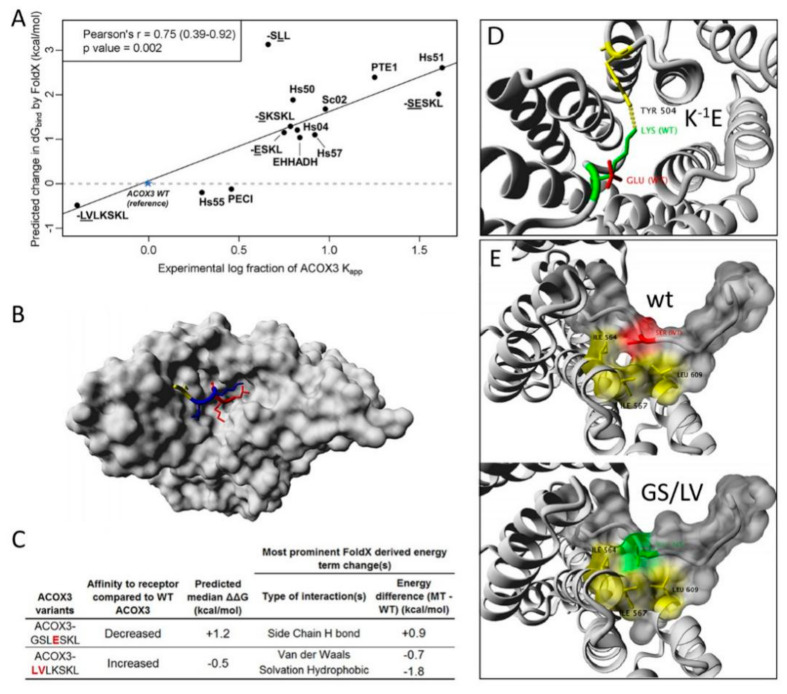
Computational investigation of the interaction between the TPR domain of PEX5 and PTS1 containing peptides: (**A**) The computationally calculated binding strengths between PEX5^TPR^ and different PTS1 peptides described in Figure 3A and Figure 5A,H correlates with K_a_^app^ values obtained by FlowFRET. The binding strength is predicted by FoldX based on the 3D-structure of human PEX5^TPR^ bound to a PTS1 [43] and expected differences upon replacing the peptide are calculated, which are expressed as change in the binding energy ΔΔG relative to a reference peptide (ACOX3). The K_a_^app^s obtained by FlowFRET experiments are depicted as logarithm of the ratio between the K_a_^app^ of the reference peptide (ACOX3) and the K_a_^app^ of the peptide of interest. Variants of ACOX3 are indicated by the peptide sequence underlining the introduced residues; (**B**) 3D model of PEX5^TPR^ burying the PTS1 of ACOX3 with the C-terminal tripeptide (red), the residues −1 and −2 (blue) and the upstream sequence −3 and −4 (yellow); (**C**) prediction of the predominant type of energetic change upon introduction of mutations (red) using FoldX; (**D**,**E**) putative rearrangement of side chains at the interphase upon introduction of the mutations: the point mutation K^-1^E ablates the hydrogen bond to tyrosine (Y504) (**D**), whereas the double mutation ^-4^GS^-3^/LV introduces a hydrophobic surface facilitating an additional hydrophobic interaction with PEX5 (red: serine, green: valine, (**E**)).

The modulatory influence of upstream sequences was originally linked to an exposing function for the C-terminal tripeptide, but its direct involvement in PEX5 binding was equally plausible. A computational approach using the FoldX suite [42], which calculates the binding energy based on the 3-dimensional–structure of the crystallized complex, allows the exclusive consideration of the final state of a binding process (Supplementary Text S14.3.9.). First, we evaluated whether such calculations based on the 3D-structure of PEX5^TRP^ interacting with the pentapeptide -YQSKL (PDB:1FCH) [36] can recapitulate differences in the interaction strength of peptide variants harboring mutations in the last three residues, which had been determined by a peptide binding assay [17]. This comparison showed a high correlation, which was not markedly deteriorated when using another 3D-structure of PEX5^TPR^ interacting with full length sterol carrier protein 2 (SCP2) (PDB:2C0L) [43] or when substituting SCP2 with different peptides within the latter structure (Supplementary Figure S11). Next, we used the same algorithm to predict the interaction strength between PEX5^TPR^ and the peptides investigated in our study. The predicted changes in binding strength are reflected by changes in the release of Gibb’s energy upon binding (change in dG_bind_; ΔΔG) using the PTS1 of ACOX3 as internal reference (Figure 6A and Supplementary Figure S11). Analogously, the experimentally determined interaction strength of the peptides was depicted as logarithm of the ratio between the K_a_^app^ of the respective peptide and the K_a_^app^ of ACOX3. The changes in the predicted binding strength showed a strong correlation with those obtained by FlowFRET (Figure 6A), which suggests that the computational method properly reflects the interaction in living cells, although it exclusively considers the interface between PEX5 and the peptides.

As the majority of the peptides depicted in Figure 6A terminate in “-SKL” (more specifically all except Hs55, Hs57, Hs50 and Hs51, cf. Figure 3A and Figure 5A,H), the differences in binding strength can be retraced to the upstream region of the PTS1, which reveals a marked contribution of this sequence to the direct interaction strength between PEX5 and PTS1. Some interesting details are that the PTS1 of EHHADH is also predicted with higher affinity than that of PTE1 (cf. Figure 5C) and that for ACOX3-peptides the additive effect of combining two point mutations (^-1^K/E and ^-2^L/S), but also the higher affinity of the ^-4^GS^-3^/LV variant were correctly predicted. However, FoldX also retraces changes in the binding strength to different thermodynamic contributions and predicts a reorientation of side chains at the interphase (Figure 6B). The mutation ^-1^K/E is predicted to lack H-bond-mediated energy (Figure 6C), which is retraced to the loss of a critical H-bond between the lysine in the PTS1 (K-1, green) and a tyrosine of PEX5 (Y504) (Figure 6D). In contrast, the introduction of hydrophobic residues by the double mutation ^-4^GS^-3^/LV is predicted to raise the hydrophobic contribution to the binding strength (Figure 6C), which is in line with an increase in the hydrophobic interphase between this PTS1 peptide and three hydrophobic residues of PEX5 (Ile564, Ile567 and Leu609) (Figure 6E). Overall, the forces at the interaction surface have a high explanatory power for the experimentally determined differences in the interaction strength between the PTS1 variants and PEX5^TPR^ in living cells.

## 4. Discussion

In this manuscript, we describe the extensive validation of a novel approach to study PPI within living cells by FRET efficiency measurements studying the interaction between PTS1 and PEX5^TPR^. Similar to standard biochemical methods, this approach provides quantitative values for the interaction strength, but the measurements are performed in the microenvironment of living cells. This can be highly important as the presence of additional interaction partners, molecular crowding and a high viscosity may affect PPIs markedly, which can hardly be mimicked in an artificial environment. Moreover, the presence of specific types of proteins such as chaperones, which specifically interact with characteristic regions of proteins (e.g., hydrophobic patches), can affect some interactions, but cannot be replicated by chemical mimics of molecular crowding (such as PEG). For example, the PTS1 of EHHADH binds better to PEX5 than that of PTE1, whereas in the peptide binding assay the results were the reverse (cf. Figure 5A,C) [16].

DFRET is the normalized measure of apparent FRET efficiency in individual cells and reflects the fraction of donor molecules involved in a complex. Thus, plotting DFRET against the acceptor to donor ratio displays a saturation curve, and K_a_^app^ and FRET_max_ values become extractable by a fitting algorithm, which is particularly powerful for large data sets obtained by flow cytometry (FlowFRET). Using FRET measurements to determine the fraction of bound and free molecules and to extract physical parameters has been suggested previously [44,45,46], but often used the traditional saturation curve, whereas the application of normalized intensities and DFRET directly in the law of mass action allows a proper consideration of the variability of both donor and acceptor. Accordingly, alternative calculations of measures of FRET based on different models, which do not predict the situation in living cells to the same degree [45,47], are certainly valid, but cannot provide the form of data, which is compatible with our fitting, as has been discussed earlier [30].

The prediction of K_a_^app^ values was found insensitive to differences in the level of FRET_max_ or to the total concentration of donor and acceptor molecules (see Figure 2). The latter was an important control for our experiment, but in other cases the complex cellular environment may actually affect the apparent interaction strength in a concentration dependent manner. Exemplarily, an endogenous stabilizing factor of a larger complex might be limiting or endogenous proteins could act as competitors. Applying this method systematically to the PTS1-PEX5 interaction, we demonstrate its high reproducibility and its ability to discriminate interaction strengths covering several orders of magnitude. Moreover, the predicted K_a_^app^ values correlate with results obtained by in vitro measurements and reporter gene-based assays. It is important to note that K_a_^app^ must not be confused with the thermodynamic measure of affinity (K_a_), although both measures are proportionally related. The huge numerical difference between the K_a_ (= 1/K_d_) of in vitro binding experiments and our results originates from the numerical difference between low-molar concentrations of cellular proteins and the high output values in fluorescence intensity measurements. In addition, we demonstrate the broad application range of our method by introducing competition experiments in living cells. The additional expression of Cerulean–PTS1 effectively reduced the apparent interaction strength between mCherry-PEX5^TPR^ and EGFP–PTS1 in a dose-dependent manner. Accordingly, in cell populations with similar acceptor-to-donor ratios, an increase in competitor-to-donor ratio is accompanied by a reduction in DFRET values, as is the predicted K_a_^app^ extracted from subpopulations sharing the same competitor-to-donor ratio. The slope of these decay curves reflects the relative affinity of donor and competitor for the acceptor, independently of whether the competing peptides have high (ACOX3 variants) or low (Hs55 versus Hs57) sequence similarity. The presence of Cerulean-based competitor proteins does not interfere with FRET-measurements between EGFP and mCherry, because Cerulean is excited at a shorter wavelength, but allows the independent determination of competitor level. Moreover, FRET effects between donor (EGFP-PTS1) and competitor (Cerulean-PTS1) need not be considered, because these proteins do not interact, whereas the underestimation of the competitor level due to a FRET between Cerulean and mCherry is expected to be small (<10% of the signal even upon complete saturation of competitor with acceptor). Finally, our results demonstrate a strong modulatory effect of the upstream sequence on the binding strength between PEX5 and PTS1-carrying proteins in living mammalian cells, which is often attributed exclusively to the C-terminal tripeptide. We found that PEX5^TPR^ binds PTS1-carrying proteins sharing the same C-terminal tripeptide, but differing in the upstream sequence, with drastically diverse affinities. Moreover, even individual point mutations in the upstream sequence of a PTS1, such as K^-1^E or S^-2^L, can markedly reduce the binding strength and these effects are additive. This confirms predictions from analyzing statistically picked PEX5-binding peptides from a library [12] or naturally occurring PTS1 motifs [13,48], in which an overrepresentation of positive charges and the underrepresentation of negative charges in the upstream sequence have been observed. However, in our study, changes in the affinity can be traced back to substitutions in the upstream sequence of a PTS1 up to seven residues before the stop codon. Thus, the results complement previous studies using peptide binding or yeast-2H assays, with FlowFRET experiments being much closer to the cellular context, in which the interaction normally occurs. Our computational analyses extend these empirical observations by suggesting changes in the interaction strength based on a predicted reorientation of side chains upon the introduction of point mutations. Thus, the strong correlation between the predicted differences in the affinity and the experimentally determined differences in K_a_^app^ values strongly suggests that the modulatory role of the upstream sequence is exerted by a direct contribution to the binding strength at the interphase between PEX5 and the PTS1. Moreover, the upstream sequence of the investigated PTS1 is sufficiently flexible, as the K_a_^app^ values obtained in our approach also correlate with the affinities of the flexible peptides [16] indicating a proper exposition of the C-terminal tripeptide from EGFP. Thus, our study demonstrates two important aspects concerning the upstream sequence, namely a direct involvement in PEX5 binding as suggested by 3D structures of cargo-loaded PEX5 [36,43,49], and the proper exposure of the C-terminal tripeptide as suggested by the high abundance of residues mediating flexibility [5,13], specifically in the context of living mammalian cells. In particular, the latter aspect cannot be covered by peptide binding assays using short peptides of different length (4–8 amino acids) [16,18,19]. Considering these results, any characterization of PTS1 as sole C-terminal tripeptide should be abandoned and substituted by a more complex description including different sequence elements contributing to the quality of this type of PTS. Altogether, these results demonstrate the power of FlowFRET for the quantitative analysis of PPI in the natural environment of living cells. 

## Supplementary Materials

The following are available online at https://www.mdpi.com/2073-4409/9/11/2381/s1, Figure S1: Acceptor bleaching of the mCherry-EGFP fusion protein, Figure S2: Flow cytometry based measurement of FRET, Figure S3: Determination of correction factors for normalization, Figure S4: Independent fitting of cell populations expressing different overall levels of donor and acceptor proteins, Figure S5: Analysis of the effect of the linker domain, Figure S6: Investigation of the interaction between PEX5^TPR^ and different PTS1 variants, Figure S7: Systematic comparison of results from the investigation of the interaction between PEX5 and diverse PTS1, Figure S8: Competition experiments using either Cerulean-PTS1 (weak, Hs57) or Cerulean alone, Figure S9: Primary data for the experiments described in Figure 5, Figure S10: Primary data for the competition experiments described in Figure 5, Figure S11: Benchmarking, Table S12: Table of oligonucleotides used in this study, Table S13: Table of plasmids used in this study, Text S14: Extended Materials and Methods.

## Abbreviations

DFRET	donor-normalized FRET
FRET	Förster-resonance-energy-transfer
FRET_max_	plateau level of DFRET
K_a_^app^	apparent affinity
PPI	protein–protein interaction
PEX	Peroxin
PTS	Peroxisomal targeting signal
TPR	tetratricopeptide
z	stoichiometry factor of the complex
