# Supplementary material for: A Novel FRET Approach Quantifies the Interaction Strength of Peroxisomal Targeting Signals and Their Receptor in Living Cells
Source: Cells. 2020 Oct 30;9(11):2381. doi: 10.3390/cells9112381 (PMC7693011; doi:10.3390/cells9112381)
Supplement: Supplementary file 1 [file cells-09-02381-s001.pdf]

cells

MDPI

Supplementary Figures

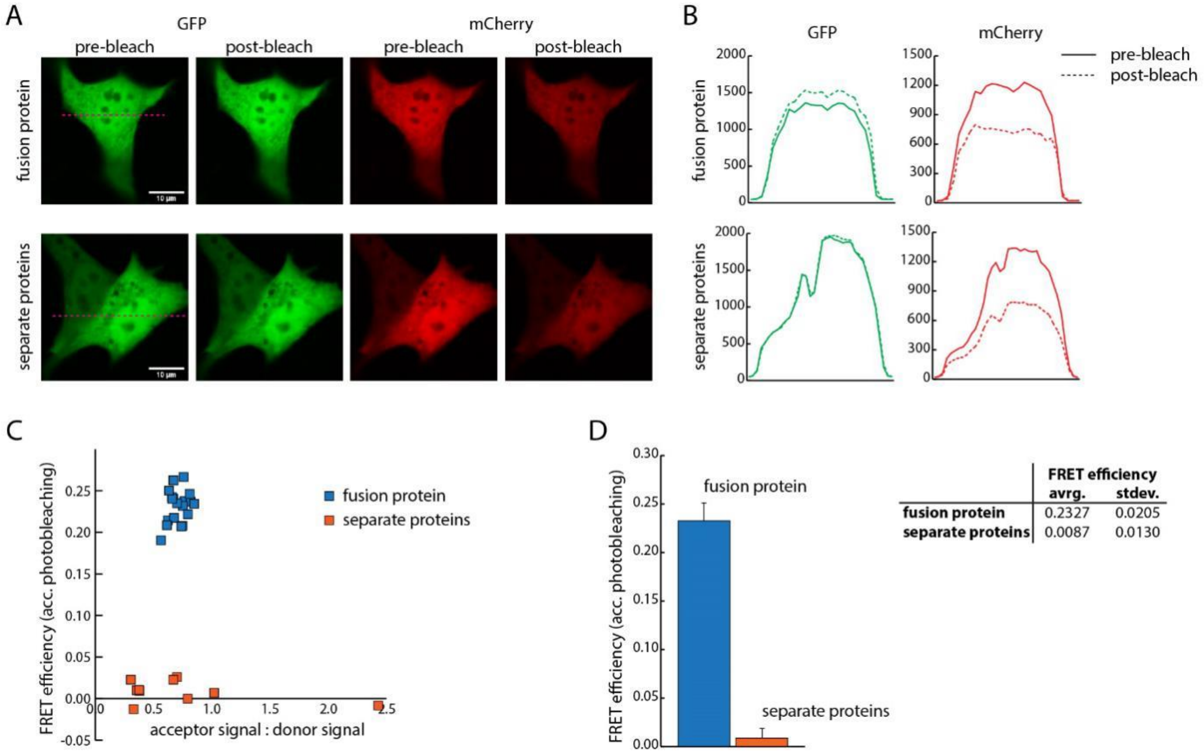

Figure S1. Acceptor bleaching of the mCherry–EGFP fusion protein: (**A**) In pex5$^{-/-}$ cells expressing either the mCherry–EGFP fusion protein or mCherry and EGFP as independent proteins, the fluorophore of mCherry is destroyed by prolonged strong excitement and the effect on EGFP–emission is detected. (**B**) Line scans for the emission of EGFP (green) and mCherry (red) before (continuous line) and after (dotted line) photobleaching, (**C**) FRET efficiency in cells expressing the mCherry–EGFP fusion protein or each protein individually, (**D**) quantification of FRET efficiency.

A

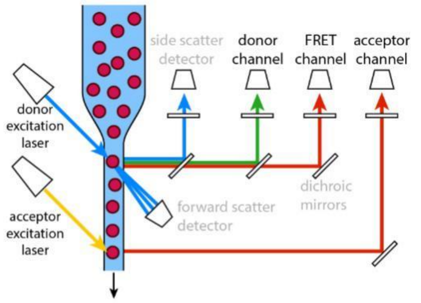

B

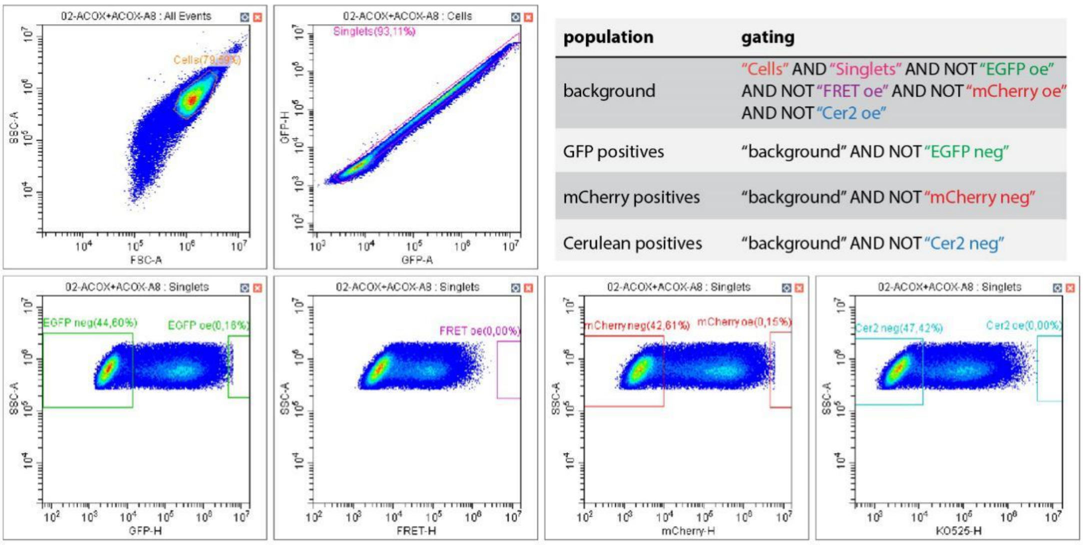

population	gating
background	"Cells" AND "Singlets" AND NOT "EGFP oe" AND NOT "FRET oe" AND NOT "mCherry oe" AND NOT "Cer2 oe"
GFP positives	"background" AND NOT "EGFP neg"
mCherry positives	"background" AND NOT "mCherry neg"
Cerulean positives	"background" AND NOT "Cer2 neg"

Figure S2. Flow cytometry based measurement of FRET: (**A**) Schematic depiction of the cytometer with a variety of different excitation lasers and detection systems to attribute a set of individual intensities in donor, acceptor and FRET channels to a large number of cells. (**B**) Gating strategy: Cells were measured on a CytoFlex S in 6 different channels: FSC (forward scatter) and SSC (side scatter) were used to determine cell populations. EGFP (donor channel), FRET (FRET channel) and mCherry (acceptor channel) were used for calculation of concentrations and DFRET. Cer (competitor channel) was only used in competition experiments to calculate the concentration of competitor. The ratio of measured peak height and peak area was used to exclude aggregates of multiple cells. Overexposure and negative cells were gated out in height dimension prior to further calculations.

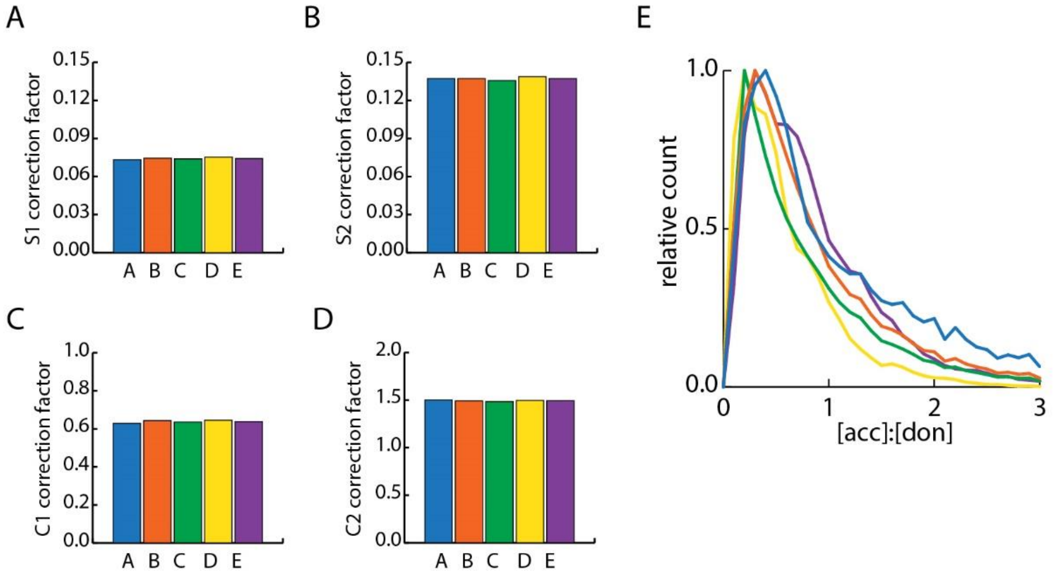

Figure S3. Determination of correction factors for normalization: The correction factors for bleed-through (**A,B**) and for the two correction factors for the donor-related (**C**) and acceptor-related (**D**) deviations in 3–filter FRET experiments were determined independently in five experiments with slightly varying populations of cells expressing different levels of donor and acceptor proteins (**E**).

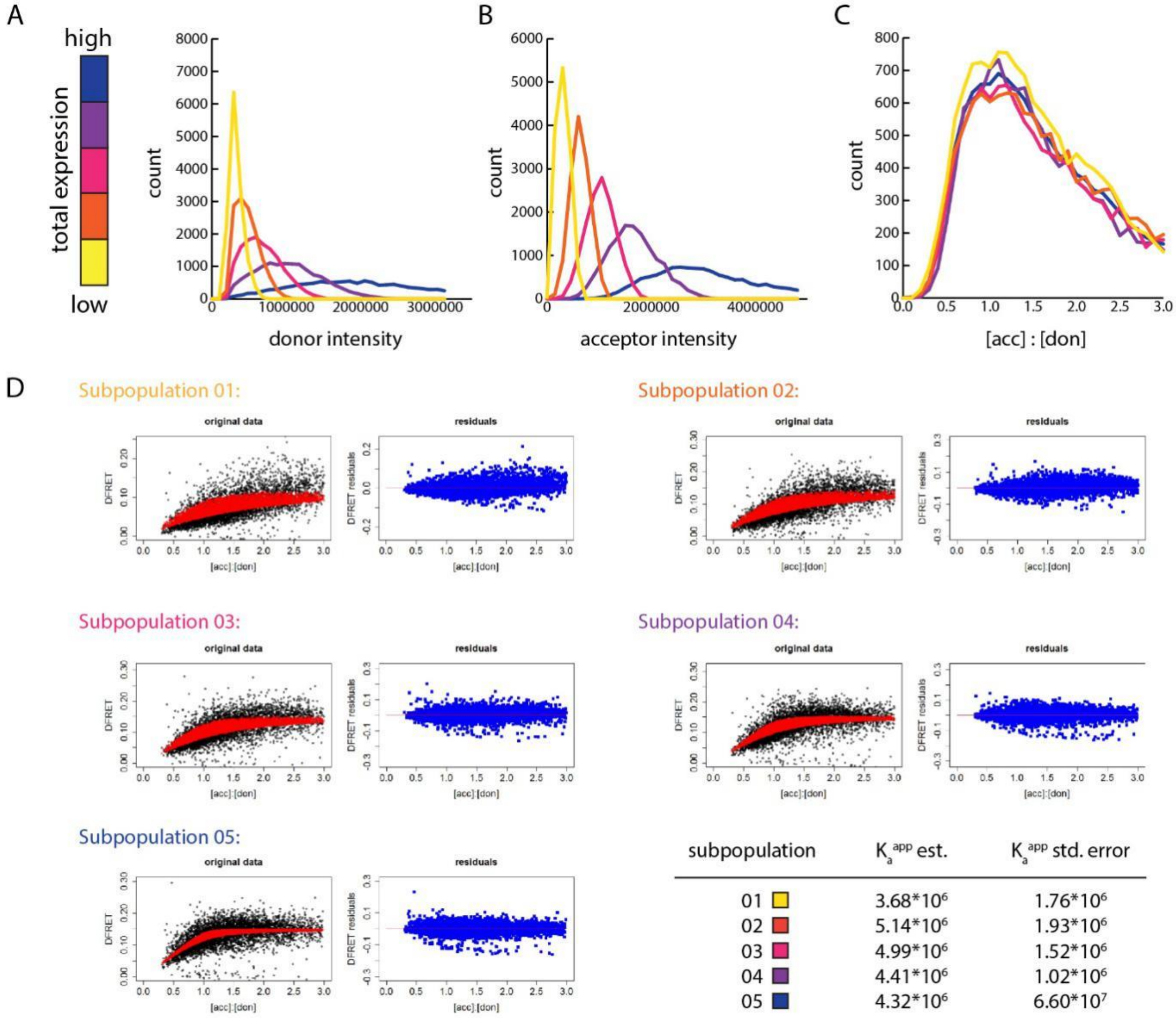

subpopulation	K_a^{app} est.	K_a^{app} std. error
01	3.68*10^6	1.76*10^6
02	5.14*10^6	1.93*10^6
03	4.99*10^6	1.52*10^6
04	4.41*10^6	1.02*10^6
05	4.32*10^6	6.60*10^7

Figure S4. Independent fitting of cell populations expressing different overall levels of donor and acceptor proteins: The entirety of cells (*cf.* Figure 2**B**) was subdivided into subpopulations expressing different levels of donor (**A**) or acceptor proteins (**B**), but present with a comparable pattern of cells according to the acceptor-to-donor ratio (**C**). FlowFRET results of these cell populations are depicted (**D**) and the numerical output of the fitting algorithm for each population is specified. Black dots represent original data points, red dots are the sated points ordered according to the formula using the extracted parameters for apparent interaction strength (K_a^{app}), the stoichiometry factor (z) and the plateau-level of the saturation curve ($FRET^{max}$).

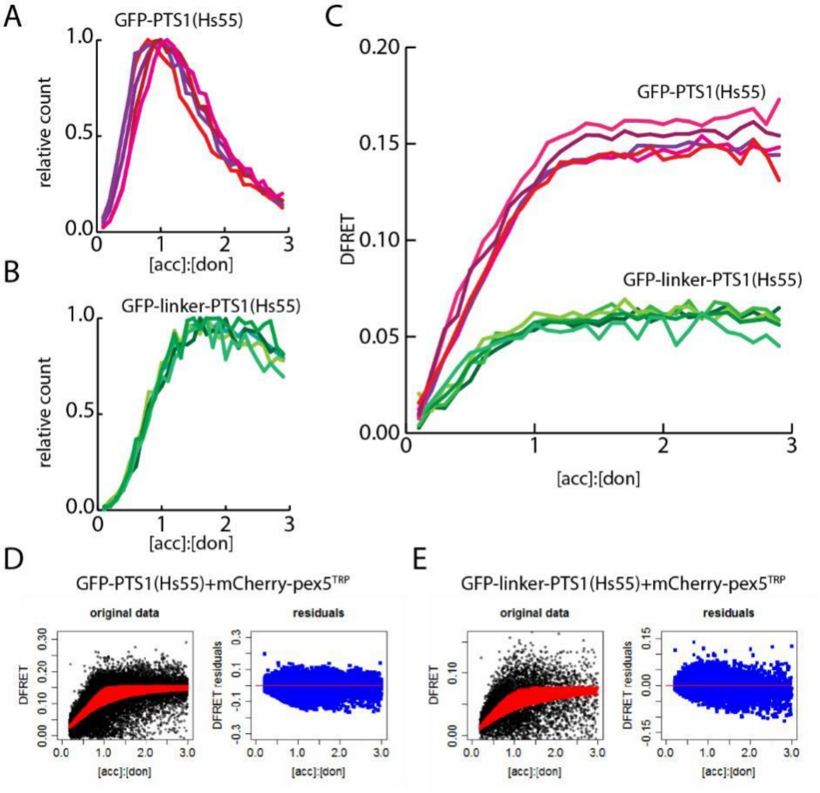

Figure 5. Analysis of the effect of the linker domain: Five independent FlowFRET experiments for cells expressing either the reference protein (EGFP–PTS1 (Hs55)) or the protein including the linker (EGFP–linker–PTS1 (Hs55)) with very similar ratios of donor and acceptor proteins (**A**,**B**) but markedly different $FRET_{max}$ values (**C**). Clouds of data points are depicted (**D**,**E**).

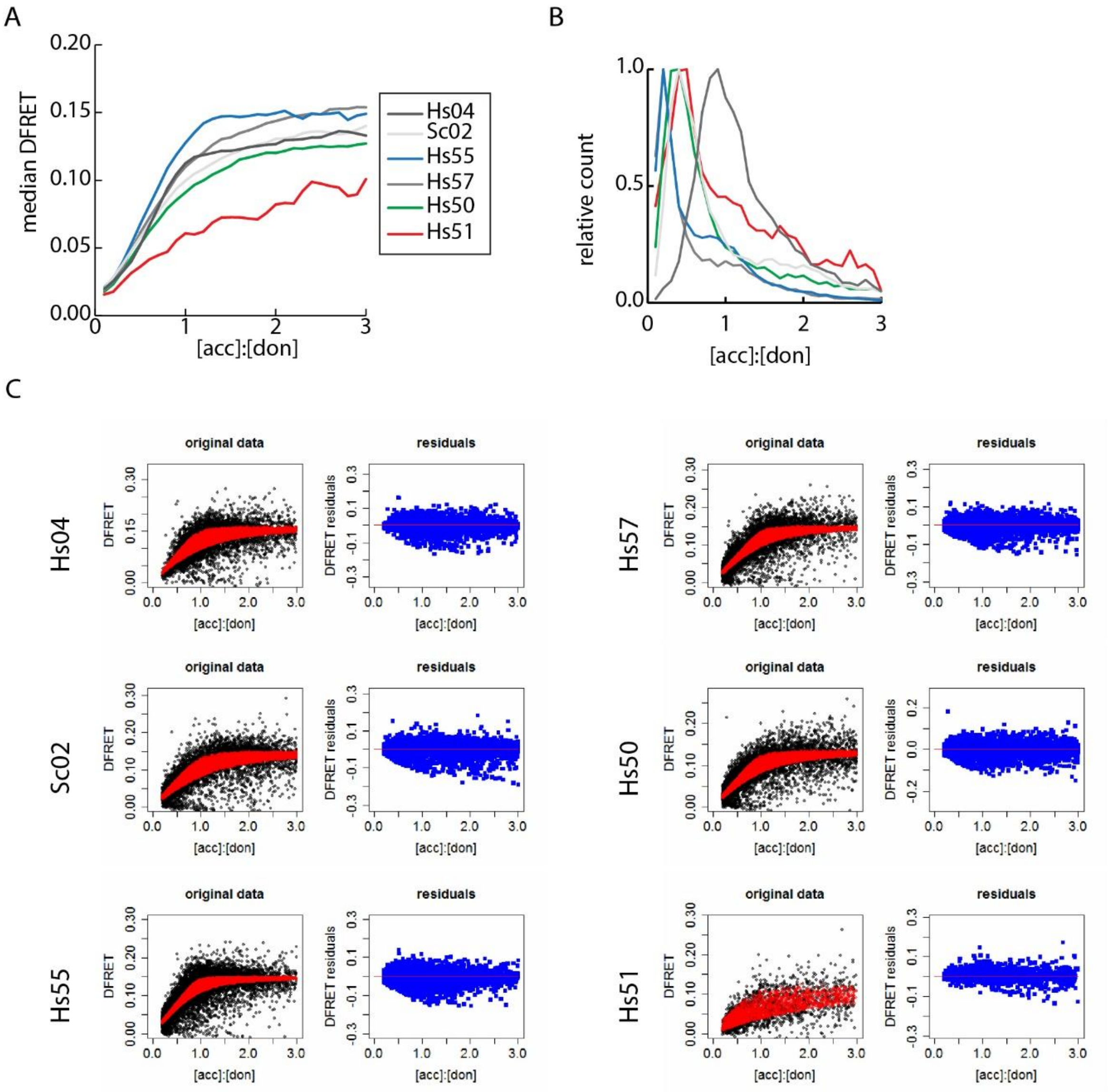

Figure S6. Investigation of the interaction between PEX5^{TPR} and different PTS1 variants: The complete data sets obtained for the FlowFRET measurements using six different PTS1 peptides (*cf.* Figure 3); the moving medians of all six curves (**A**), the distribution of cells with differing acceptor-to-donor ration (**B**) and the FRET-analysis of sorted cells (**C**) are depicted.

.

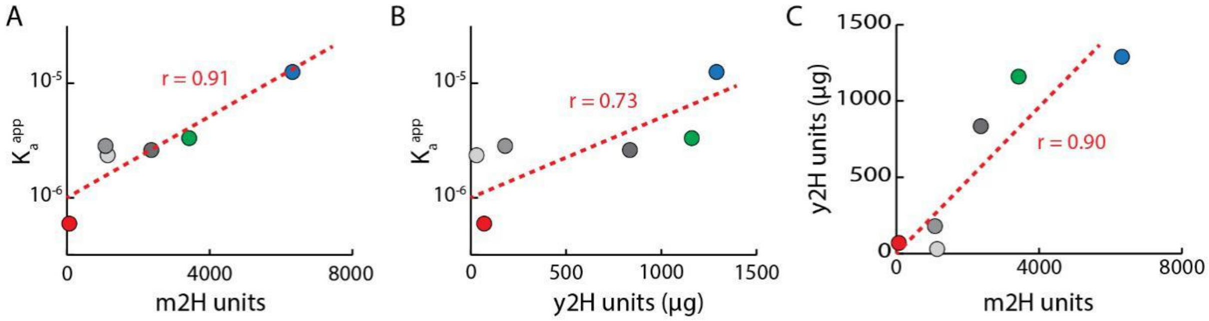

Figure S7. Systematic comparison of results from the investigation of the interaction between PEX5 and diverse PTS1: Correlation between the results obtained by FlowFRET and the mammalian two hybrid (2H)–assay (**A**), FlowFRET and the yeast two–hybrid assay (**B**), and the mammalian and the yeast–2H–assay (**C**).

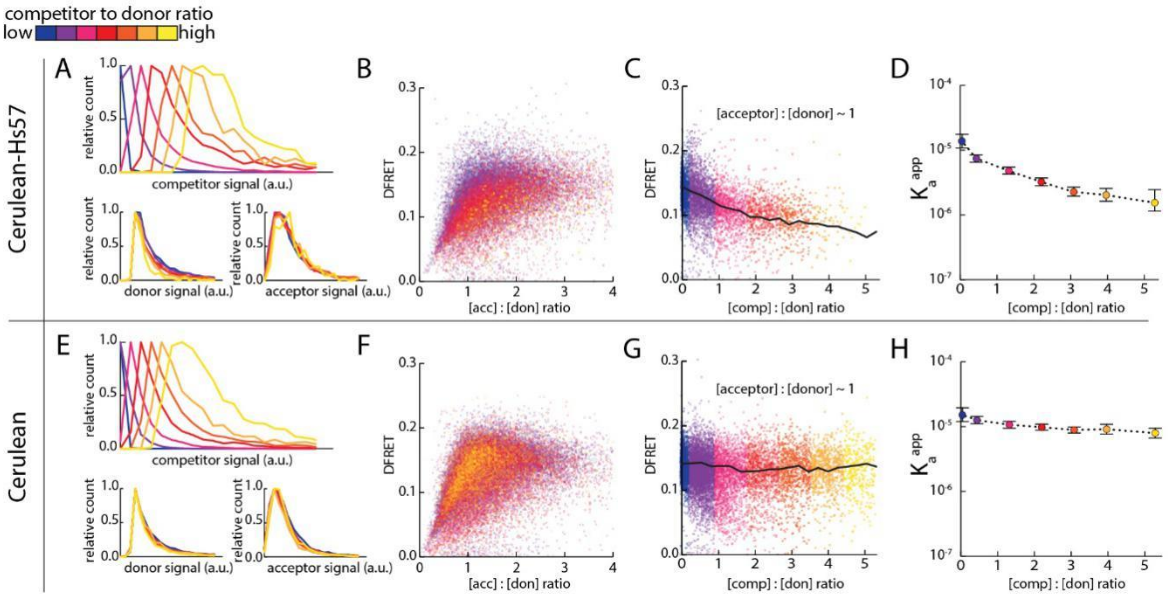

Figure S8. Competition experiments using either Cerulean–PTS1 (weak, Hs57) or Cerulean alone: Details for the FlowFRET experiment described in Figure 4**G–J** highlighting the distribution of cells with specified competitor, donor or acceptor signal (**A,E**), a plot of DFRET against the acceptor-to-donor ratio (**B,F**), the decay DFRET with increasing competitor to donor ratios of subpopulations with acceptor-to-donor ratios around 1 (**C,G**) and the decay of K_a^{app} upon increasing competitor to donor ratios, when subjecting the subpopulations described in S09a and S09e to the fitting algorithm.

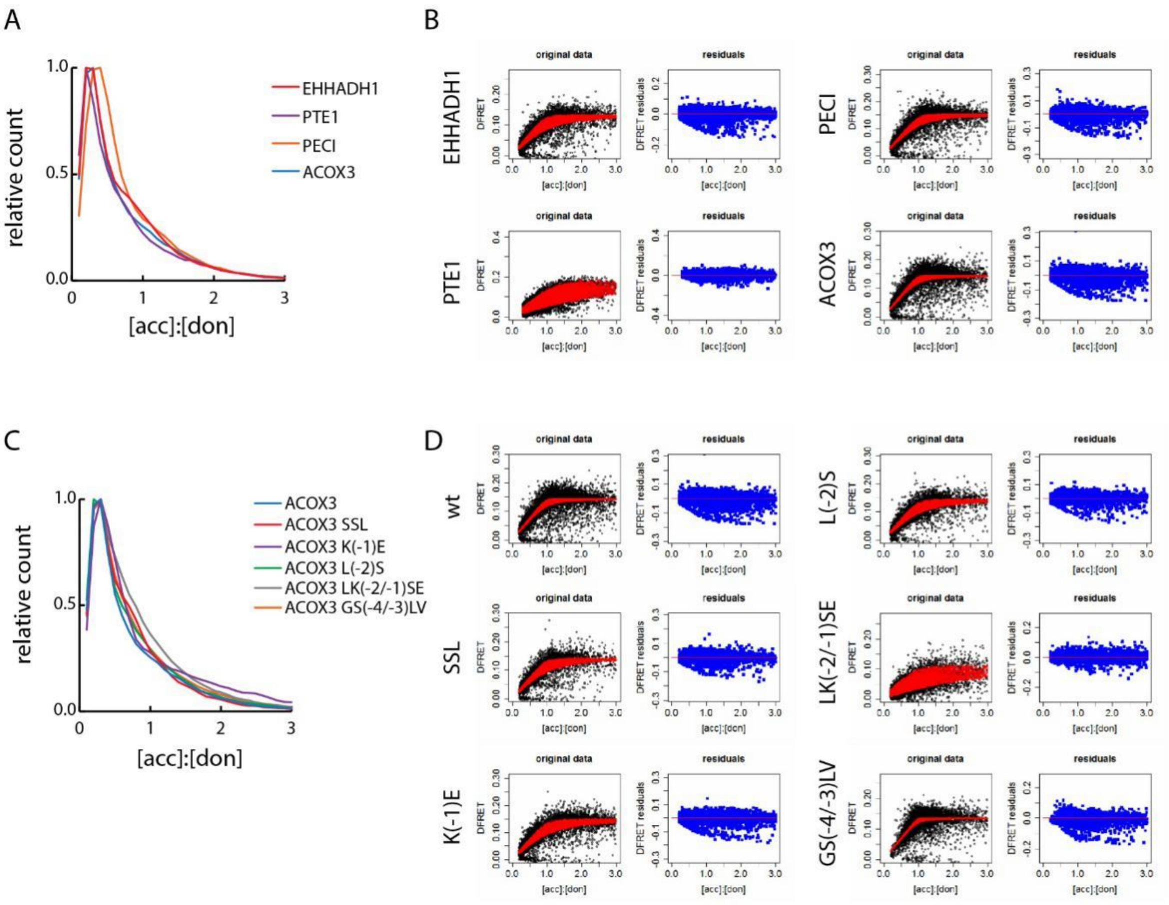

Figure S9. Primary data for the experiments described in Figure 5: The entirety of data points analyzed, the areas of the confidence intervals and the average deviation of individual residuals are depicted.

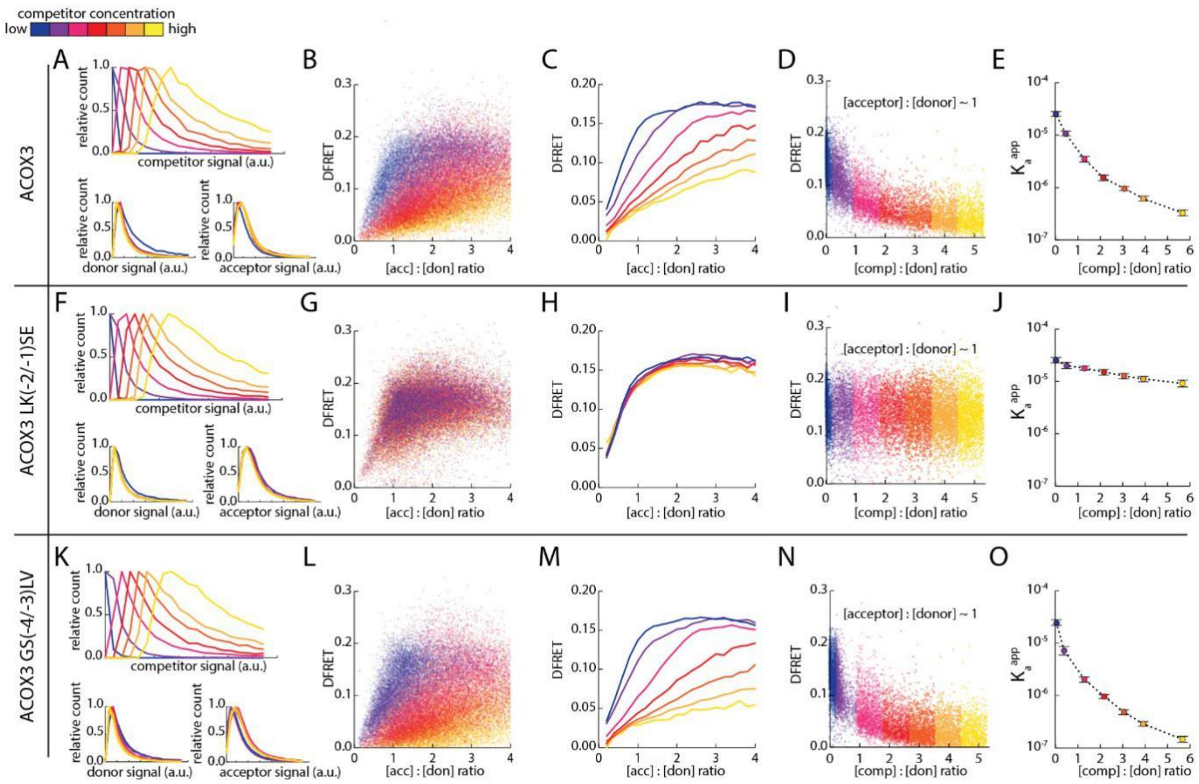

Figure S10. Primary data for the competition experiments described in Figure 5: The interaction between mCherry–PEX5^{TPR} and EGFP–PTS1 (ACOX3) is differently affected by the additional expression of Cerulean–PTS1 (ACOX3), or variants thereof harboring two point mutations Cerulean–PTS1 (ACOX3 $^{-2}$LK^{-1}/SE) and Cerulean–PTS1 (ACOX3^{-4}GS^{-3}/LV); the distribution of cells with a specified competitor, donor and acceptor expression and the color code for subpopulations differing in the competitor to donor levels (**A,F,K**), a plot of DFRET values against the acceptor-to-donor ratio for the different subpopulations (**B, G, L**) and binning the data sets along the *x*-axis allows the depiction of each of the subpopulations (**C,H,M**). Furthermore, the decay of DFRET upon increasing competitor to donor ratios for subpopulations with acceptor-to-donor ratios of around 1 (**D,I,N**) and K_a^{app} obtained for the different subpopulations by the fitting algorithm (**E,J,O**) are depicted.

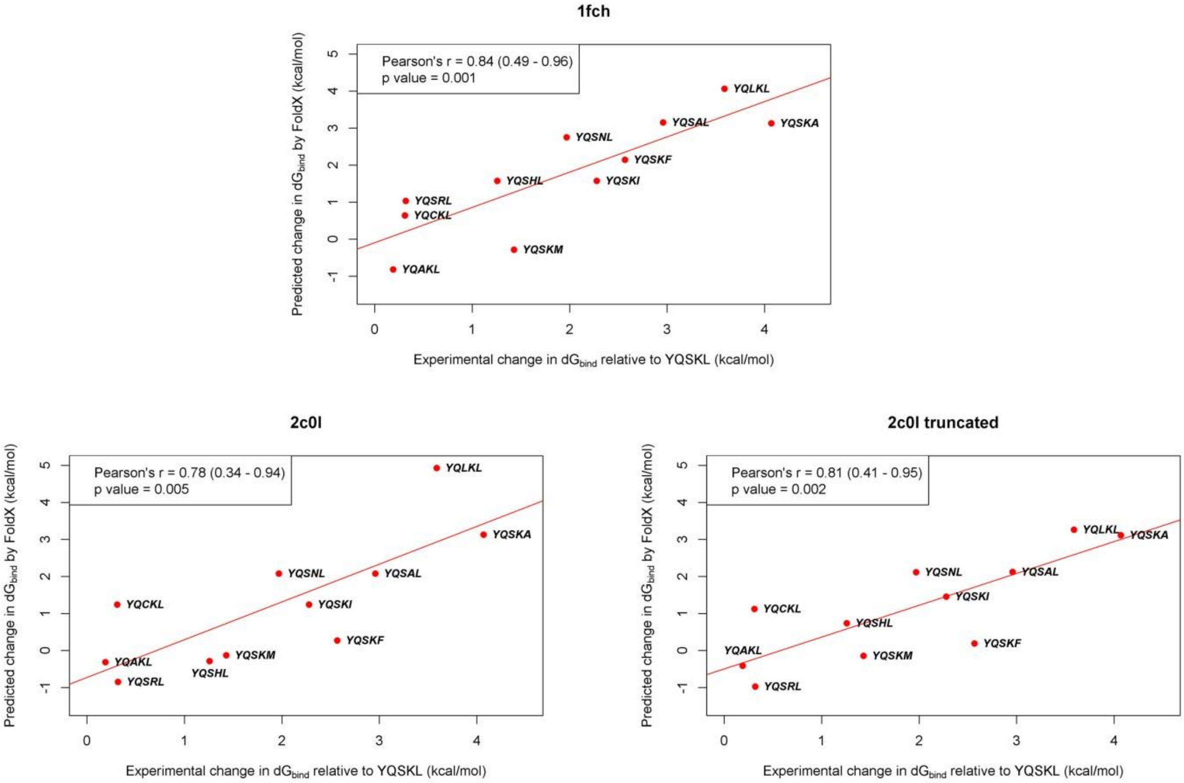

Figure S11. Benchmarking: Results of benchmarking studies with PTS1 mutants from [1]. Strong correlations were obtained between predicted and experimental binding energies ($\Delta\Delta G$). Numbers in brackets denote the 95% CI for Pearson's r.

Table S12. Table of oligonucleotides used in this study.

Oligo-nr.	Sequence	Orient.	Name	R.s.
Oli_2540	GATCTATggcaaagcttggcaggctcccctagcagtaaattgtgaA	fw	EHHADH1	BglII
Oli_2541	AGCTTtcacaatttactgctaggggagcctgccaagctttgccATA	rev	EHHADH1	HindIII
Oli_2542	GATCTAAtccgagtgaagccccaggtctcagagagcaagctgtagA	fw	PTE1	BglII
Oli_2543	AGCTTctacagcttgctctctgagacctggggcttcactcggaTTA	rev	PTE1	HindIII
Oli_2544	GATCTAaacaaacctgtcataggaagtctgaaatcgaagctctagA	fw	ACOX3	BglII
Oli_2545	AGCTTCtagagcttcgatttcagacttcctatgacaggtttgttTA	rev	ACOX3	HindIII
Oli_2546	GATCTAgctgtggtgaacttcttatccagaaaatcaaaactgtgaA	fw	PECI	BglII
Oli_2547	AGCTTtcacagttttgattttctggataagaagttcaccacagcTA	rev	PECI	HindIII
Oli_2548	GATCTAAacaaacctgtcataggaagtctgaaatcgCTGctctagA	fw	ACOX3 (SLL)	BglII
Oli_2549	AGCTTctagagCAGcgatttcagacttcctatgacaggtttgtTTA	rev	ACOX3 (SLL)	HindIII
Oli_2550	GATCTAAacaaacctgtcataggaagtctgGAAtcgaagctctagA	fw	ACOX3 (K-1E)	BglII
Oli_2551	AGCTTctagagcttcgaTTCcagacttcctatgacaggtttgtTTA	rev	ACOX3 (K-1E)	HindIII
Oli_2552	GATCTAAacaaacctgtcataggaagtTCGaaatcgaagctctagA	fw	ACOX3 (L-2S)	BglII
Oli_2553	AGCTTctagagcttcgatttCGAacttcctatgacaggtttgtTTA	rev	ACOX3 (L-2S)	HindIII
Oli_2554	GATCTAAacaaacctgtcataggaagtTCGGAAtcgaagctctagA	fw	ACOX3 (LK/SE)	BglII
Oli_2555	AGCTTctagagcttcgaTTCCGAacttcctatgacaggtttgtTTA	rev	ACOX3 (LK/SE)	HindIII
Oli_2556	GATCTAAacaaacctgtcataCTGGTActgaaatcgaagctctagA	fw	ACOX3 (GS/LV)	BglII
Oli_2557	AGCTTctagagcttcgatttcagTACCAGtatgacaggtttgtTTA	rev	ACOX3 (GS LV)	HindIII
Oli_2567	GTACGGCGGTGGATCCGGAGGTAGCGGAGGTAT	fw	Linker	BsrGI
Oli_2568	GTACATACCTCCGCTACCTCCGGATCCACCGCC	rev	Linker	BsrGI

R.s., restriction site; Orient., orientation

Table S13. Table of plasmids used in this study.

Number	Content	Aim	Description
#2003	mCherry–EGFP	Positive control	this study
#1932	mCherry–PEX5 (TPR)	Acceptor	this study
#1993	mCherry–PEX5 (TPR) N526K	Acceptor	this study
#127	EGFP	Donor	Clontech
#126	EGFP–PTS1 (Lametschwandtner, 1998) (Hs04)	Donor	Lametschwandtner, 1998
#2036	EGFP–PTS1 (Hs50) (1H1A)	Donor	Lametschwandtner, 1998
#2037	EGFP–PTS1 (Hs04) (3H45)	Donor	Lametschwandtner, 1998
#2038	EGFP–PTS1 (Hs57) (FD11)	Donor	Lametschwandtner, 1998
#2039	EGFP–PTS1 (Sc02) (2/9B)	Donor	Lametschwandtner, 1998
#2057	EGFP–PTS1 (Hs55) (Hs3H25)	Donor	Lametschwandtner, 1998
#2058	EGFP–PTS1 (Hs51) (1FX)	Donor	Lametschwandtner, 1998
#2299	EGFP–PTS1_linker (Hs55)	Donor	this study
#2263	EGFP–PTS1 (ACOX3) (peptide 1)	Donor	this study
#2264	EGFP–PTS1 (PECI) (peptide 2)	Donor	this study
#2262	EGFP–PTS1 (PTE1) (peptide 3)	Donor	this study
#2261	EGFP–PTS1 (EHHADH1) (peptide 4)	Donor	this study
#2271	EGFP–PTS1 (ACOX3-SSL) (peptide 5)	Donor	this study
#2272	EGFP–PTS1 (ACOX3- K(–1)E) (peptide 6)	Donor	this study
#2265	EGFP–PTS1 (ACOX3 L(–2)E) (peptide 7)	Donor	this study
#2273	EGFP–PTS1 (ACOX3 LK(–4/–3)SE) (peptide 8)	Donor	this study
#2266	EGFP–PTS1 (ACOX3 GS (–5/–4)LV) (peptide 9)	Donor	this study

	**Two Hybrid plasmids**		
#1495	VP16–hPEX5(TRP)	2H plasmid	this study
#2208	pM–EGFP–SKL (original)	2H plasmid	this study
#2209	pM–EGFP–PTS1 (1FX)	2H plasmid	this study
#2210	pM–EGFP–PTS1 (FD11)	2H plasmid	this study
#2211	pM–EGFP–PTS1 (3H25)	2H plasmid	this study
#2212	pM–EGFP–PTS1 (1H1A1)	2H plasmid	this study
#2213	pM–EGFP–PTS1 (2/B9)	2H plasmid	this study
#2267	pM–EGFP	2H plasmid	this study
#2324	pM–EGFP–PTS1 (ACOX3)	2H plasmid	this study
#2327	pM–EGFP–PTS1 (ACOX3_SKL-SLL)	2H plasmid	this study
#2328	pM–EGFP–PTS1 (ACOX3-K-1E)	2H plasmid	this study
#2325	pM–EGFP–PTS1 (ACOX3_L-2S)	2H plasmid	this study
#2329	pM–EGFP–PTS1 (ACOX3_LK/SE)	2H plasmid	this study
#2326	pM–EGFP–PTS1 (ACOX3_GS/LV)	2H plasmid	this study

**Controls**			
#1965	mCherry–Cerulean	Control	this study
#2274	Cerulean–PTS1 (Hs55)	Competitor	this study
#2285	Cerulean–PTS1 (Hs57)	Competitor	this study
#2339	Cerulean–PTS1 (ACOX3)	Competitor	this study
#2341	Cerulean–PTS1 (ACOX3-LK/SE)	Competitor	this study
#2340	Cerulean–PTS1 (ACOX3-GS/LV)	Competitor	this study

Text S14. Extended Materials and Methods.

1. Cloning Procedure

Plasmid list: all plasmids used in this study are listed in Supplementary Figure 12. **Oligonucleotide list**: all oligonucleotides used are listed in Supplementary Figure 13.

mCherry–EGFP (#2003) was obtained by digesting EGFP–N3 (#384) with restriction enzymes NdeI and BglII and ligating it with a DNA fragment encoding mCherry obtained by digestion of mCherry–PEX5(TPR) with restriction enzymes NdeI and BglII.

mCherry–Cerulean (#1965) was obtained by ligating the DNA fragment encoding mCherry-C1 (#1945) excises by NdeI and SalI with Cerulean–N digested with the same enzymes. Cerulean-N has been obtained by exchanging the open reading frame (ORF) of EGFP–N1 lacking the start codon (EGFP–ΔATG; #381) [2]) with the ORF of Cerulean (Clontech) using the restriction enyzmes BsrGI and AgeI (3975).

mCherry–PEX5^{TPR} (#1932) was obtained by digesting hP87 (pGBT9-HsPEX5_TPR) [3] with EcoRI/SalI and ligating with mCherry–C1–(XhoI) (#1946) with the same enzymes. mCherry–C1–(XhoI) is derived from mCherry–C1 by digestion with XhoI, filling up of the overlaps by Klenow polymerase and relegation.

mCherry–PEX5^{TPR}_N562K (#1993): mCherry–C1–(XhoI) (#1946) was digested with EcoRI/SalI and ligated with a DNA fragment encoding the TPR-domain of PEX5 with the point mutation N526K, which has been digested with EcoRI/SalI. The PEX5 variant has been recloned from an expression plasmid encoding this mutation.

PTS1 carrying EGFP fusion proteins: EGFP–PTS1 variants (e.g., #126): EGFP–C3 (Clontech) was digested with BglII/HindIII and annealed oligonucleotide pairs were ligated [3]. EGFP–PTS1 (1FX) was obtained by digesting EGFP-C3 with restriction enzymes BglII and HindIII and ligating it with the short DNA-fragment obtained from the y2H clone 1FX digested with BamHI and HindIII (4094). The other peptides were derived by digesting EGFP–C3 with restriction enzymes BglII and HindIII and ligating it to the pre-annealed oligonucleotides XX and YY (compare to Figure S13) to obtain the proper plasmids. Insertion of the peptides between the PTS1 and the core protein: EGFP–linker–PTS1 (Hs55) was obtained by digesting plasmid EGFP–PTS1(Hs55) with the restriction enzyme BsrGI and inserting the oligonucleotides 2567 and 2568 (according to the table).

Competitive Cerulean protein: Cerulean–PTS1 (P2091): EGFP–PTS1 (#126) was digested with the restriction enzymes NdeI and BsrGI and this vector fragment was ligated to a DNA fragment obtained by digestion of Cerulean-C1 with the same enzymes, which contain part of the CMV–promoter and the ORF of Cerulean. Similar variants of Cerluean–PTS1 encoding the PTS1 or Hs55 and Hs57 were obtained by ligating the vectors obtained by digesting the EGFP–PTS1 plasmids #2057 and #2038 with restriction enzymes NheI and BsrGI and the insert obtained by digesting the plasmid #1885 (company) encoding the ORF of Cerulean with the same enzymes. Similarly, the plasmids Cerulean–PTS1 (ACOX3), Cerulean–PTS1 (ACOX3 LK/SE) and Cerulean–PTS1 (ACOX3 GS/LV) were obtained by digesting the EGFP fusion proteins with the restriction enzymes NheI and BsrGI and ligating these vectors with DNA fragments obtained by digesting the expression plasmid for Cerulean with the same enzymes (#1885). Cerulean–C1 was a gift from Michael Davidson and Dave Piston [4].

M2H plasmids: pVP16–HsPEX5^{TPR} (prey) was obtained by digesting the plasmid pGBTP9-HsPEX5(TPR) [3] with EcoRI and SalI and ligating the DNA–fragment with the vector pVP16 digested with EcoRI and SalI. pM–EGFP–SKL (original, bait) was cloned in a two-step process, first by digesting pM (1389) with BamHI/SalI and ligating it with a DNA–fragment containing EGFP–SKL (4363) and the product was cut with BamHI, then the ends were blunted by Klenow fill followed by relegation to obtain pM–EGFP–PTS1 (original, XX) (#2208). This product was further processed to obtain other plasmids encoding EGFP with other C-terminal peptides. Therefore, pM–EGFP–PTS1 was digested with AgeI and SalI and ligated to different variants of EGFP–PTS1 digested with the same enzymes, resulting in the Gal4p^{DBD}-EGFP–PTS1 expression plasmids

encoding the peptides 1FX (#2209), FD11 (2210), 3H25 (#2211), 1H1A1 (#2212) and 2/9B (#2213). Similarly, the plasmids pM–EGFP–PTS1 encoding the PTS1 of ACOX3 or variants thereof were generated by the same procedure. **pM–EGFP** was cloned by digesting EGFP–N2 (Clontech, XX) with the restriction enzymes NotI/BamHI and ligating the excised ORF of EGFP with the plasmid pM–PTS2–EGFP [5], which has been digested with the same enzymes. The plasmids encoding pM–EGFP–PTS1 with diverse ACOX3 variants were produced by digesting the corresponding EGFP–plasmids with restriction enzymes AgeI and SalI to obtain the EGFP–PTS1 ORFs and then ligating these inserts with pM–EGFP–PTS1 (1FX) digested with the same restriction enzymes.

2. Culture Conditions and Transfections

The human cell line HeLa was purchased from ATCC and murine immortalized embryonic fibroblasts (MEF) of a PEX-deficient mouse model was obtained from Prof. Myriam Baes [6]. Cells were cultivated in Dulbecco´s modified eagle´s medium (DMEM) supplemented with 10% fetal calf serum (FCS), 2 mM L–glutamine, 50 U/mL penicillin and 100 µg/ml streptomycin (Biowhittaker). Cells were transfected using Turbofect (Thermo-Scientific) according to the manufacturer´s instructions with a subsequent centrifugation step (swing our rotor, 300 g, 30 min) to increase transfection efficiency. For *microscopy*, $2*10^4$ cells were seeded into an 8-well glass bottom slide (ibidi, GER) and on the next day cells were incubated with a mixture of 0.5 µg DNA and 1 µl Turbofect according to the manufacturer specifications. Microscopy was done 24 to 36 h after transfection. For flow cytometry, $5*10^4$ cells were seeded into each well of a 24 well plate and on the next day cells were incubated with a mixture of 1.0 µg DNA and 2 µl Turbofect according to the manufacturer specifications, with a subsequent centrifugation step as described above. Flow cytometry was done 24 to 36 h after transfection. For flow cytometry, cells were washed with PBS once and detached with 150 µl of Trypsin/EDTA solution (Gibco) for 10 min. Detached cells were transferred into 96 well plates with V–bottom and cells were pelleted by centrifugation at 300 g for 3 min. Supernatant was removed and cell pellets were re-suspended in 150 µl PBS. Cells were immediately measured on the flow cytometer.

Mammalian two hybrid assay: 24 h after seeding $Pex5^{-/-}$ cells in 24 well plates, they were co-transfected with the luciferase and the β–galactosidase reporter plasmids pFR–Luc (0.1 µg) and pCMV–β–Gal (Promega) (0,05 µg) together with the bait (pM: $GAL4^{DBD}$ based plasmids withEGFP–PTS or pM–EGFP–PTS1) (0,35 µg) and the prey (pVP16: HSV–$VP16^{AD}$ based plamids with $PEX5^{TPR}$) (0,35 µg) plasmids using Turbofect (Fisher-Scientific, 2 µl) with a subsequent centrifugation step as described above. Approximately 48 h after transfection, cells were washed and lysed with 50 µl lysis buffer (0.1 M KH_2PO_4 + 0.1% triton + complete protease inhibitor cocktail (Roche-diagnostics, CH). After 15 min incubation, the supernatant was transferred into an Eppendorf tube and centrifuged for 15 min at 15.300 g in a table-top centrifuge at 4 °C. A total of 20 µl was transferred into a white 96 well plate and 50 µl assay buffer (20 mM $MgSO_4$, 20mM ATP, 20 mM glycyl-glycine) were added. Luciferase activity was measured on a Synergy Reader (BioTek, USA) over a 10s integration time after injection of 50 µl injection buffer (0.25 mM Luciferin, 20 mM glycyl-glycine) and 2s of shaking. The entire content of each well was subsequently transferred to a clear 96 well plate and 50 µl CPRG (chlorophenol red-beta-D-galactopyranoside) solution (2,3 mM CPRG in PBS + 0.5% BSA) were added. B–galactosidase activity was measured multiple times after 30–300 min in a Viktor–1420 spectrometer at 600 nm. Results from β–galactosidase measurements were used for normalization of transfection efficiency.

3. flowFRET Measurements

3.1. Microscopy

Microscopy was done on a Nikon A1 confocal laser scanning microscope (NIKON) system with a 60 × oil immersion objective (NIKON, Plan Apo, NA1,4). Intensity in the donor channel was

acquired at an excitation of 488 nm and emission band of 525/50 nM (550–550 nm). Intensity in the FRET channel was acquired at an excitation of 488 nm and emission band of 595/50 (575–620 nm). Intensity in the acceptor channel was acquired at an excitation of 561 nm and an emission band of 595/50 (575–620 nm).

3.2. Flow Cytometry

Flow cytometry was done on a Cytoflex S (Beckman Coulter) flow cytometer. Exact channel set-ups can be found in the Materials and Methods section of the main text. Cells of interest were selected from the entirety of cells according to the criteria defined in Suppl.Fig.2b.

3.3. Extracting Raw Data

Microscopy images were obtained in the .tif format. Background and Cells were automatically detected and measured via our previously supplied macro for ImageJ [7] (ImageJ). In flow cytometry, cells were gated via forward and side scatter in the CytExpert software (Beckman Coulter). Overexposed data points were removed via gating along the peak height of each respective channel. Remaining data points were extracted as FCS datasets and transformed into tab delimited .txt lists via a custom code for the program R, including the flowCORE package (flowCore). Further calculations were done in Microsoft EXCEL or R, respectively.

3.4. Nomenclature

Measured and normalized signals are distinguished by subscript and superscript letters. The large letter in front describes the used fluorescent channel (*D* = donor channel, *A* = acceptor channel, *F* = Fret channel, *C* = competitor channel). The subscript letter describes the sample (*d* = sample only contains donor, *a* = sample only contains acceptor, *c* = sample only contains competitor, *da* = sample contains donor and acceptor, *ca* = sample contains competitor and acceptor, etc). The superscripted letter c indicates that signal has been corrected for bleedthrough. For example: D_{da}^{c}would be the corrected donor signal from a sample containing donor and acceptor.

3.5. Treatment and Calculation of FRET Data

The evaluation of microscopy and flow cytometry-based experiments follows the same mathematical treatment. Additional to the measured samples, each experiment included the following samples for control purposes: 1) no plasmid, 2) EGFP alone, 3) mCherry alone, 4) mCherry–EGFP fusion protein, 5) EGFP and mCherry. Background within the measured channels was determined via the empty sample and subsequently subtracted from all other samples. Spectral bleed factors S1–S4 for normal FRET experiments were calculated from the samples only containing EGFP or mCherry.

$$S_1 = \frac{F_d}{D_d}$$

$$S_2 = \frac{F_a}{A_a}$$

$$S_3 = \frac{A_d}{D_d}$$

$$S_4 = \frac{D_a}{A_a}$$

This allows calculation of corrected signals Dc, Ac and Fc.

$$D_{da}^{c} = \frac{D_{da} - S_4 * A_{da}}{1 - S_3 * S_4}$$

$$A_{da}^{c} = \frac{A_{da} - S_3 * D_{da}}{1 - S_3 * S_4}$$

$$F^{c} = F_{da} - D_{da}^{c} * S_1 - A_{da}^{c} * S_2$$

The sample containing mCherry–EGFP was used to determine correctional factors C1 and C2 in order to normalize signals in the differing channels to each other (for details, see [7]). This required a known FRET efficiency of our fusion construct, as determined by acceptor photobleaching according to [7]. This was determined to be 0.23 (Supplementary Figure 1).

$$C1 = \frac{F^{c} - E * F^{c}}{E * D_{da}^{c}}$$

$$C2 = \frac{D_{da}^{c} * C1 + F^{c}}{A_{da}^{c}}$$

This allows calculation of relative concentrations of donor and acceptor, molar ratios and DFRET values.

$$[don] = D_{da}^{c} * C1 + F^{c}$$

$$[acc] = A_{da}^{c} * C2$$

$$DFRET = \frac{F^{c}}{C1 * D_{da}^{c} + F^{c}}$$

3.6. FRET Competition Experiments

Additional to the above mentioned, FRET competition experiments included the following samples: 6) Cerulean alone, 7) mCherry–Cerulean fusion protein, 8) EGFP and mCherry and Cerulean. Additional spectral bleed factors are needed to account for bleeding of fluorophores into the Cerulean channel, and bleeding of Cerulean into the other channels.

$$S_0 = \frac{F_c}{C_c}$$

$$S_5 = \frac{D_c}{C_c}$$

$$S_6 = \frac{C_d}{D_d}$$

$$S_7 = \frac{A_c}{C_c}$$

$$S_8 = \frac{C_a}{A_a}$$

Corrected channels have to be calculated different than above, in order to obtain correct signals for further calculations:

$$C_{da}^{c} = \frac{C_{da} * (1 - S_3 * S_4) + D_{da} * (S_4 * S_8 - S_6) + A_{da} * (S_3 * S_6 - S_8)}{1 + S_6 * S_3 * S_7 + S_8 * S_4 * S_5 - S_7 * S_8 - S_5 * S_6 - S_3 * S_4}$$

$$D_{da}^{c} = \frac{C_{da} * (S_3 * S_7 - S_5) + D_{da} * (1 - S_7 - S_8) + A_{da} * (S_5 * S_8 - S_3)}{1 + S_6 * S_3 * S_7 + S_8 * S_4 * S_5 - S_7 * S_8 - S_5 * S_6 - S_3 * S_4}$$

$$C_{da}^{c} = \frac{C_{da} * (S_4 * S_5 - S_7) + D_{da} * (S_6 * S_7 * S_4) + A_{da} * (1 - S_5 * S_6)}{1 + S_6 * S_3 * S_7 + S_8 * S_4 * S_5 - S_7 * S_8 - S_5 * S_6 - S_3 * S_4}$$

$$F^{c} = F_{da} - C_{da}^{c} * S_0 - D_{da}^{c} * S_1 - A_{da}^{c} * S_2$$

To normalize Cerulean intensity to the other fluorophore intensities, a third correction factor C3 is needed, which is calculated from the sample containing mCherry–Cerulean, a protein that contains mCherry and Cerulean in equal amounts. As FRET between Cerulean and mCherry was detected in our plasmid (E = 14.8%), we adjusted the correction factor for this to avoid overestimation of Cerulean levels.

$$C3 = \frac{A_{ca}^{c} * C2}{C_{ca}^{c}} * (1 - DFRET_{mCherry-Cerulean})$$

Despite this correction for FRET in the fusion protein, competitor levels can still be slightly under-estimated as FRET between Cerulean-bearing competitor and mCherry–bearing acceptor can occur, thereby reducing detectable Cerulean signal. However, this was determined to be maximally 7% and far less in most cases as it depends on the binding status of competitor and acceptor.

Therefore, this third correction factor allows the additional calculation of relative Cerulean concentration and its ratio to other fluorophores.

$$[cer] = C_{da}^{c} * C3$$

3.7. Fitting Algorithm

The fitting algorithm is based on the law of mass action and is described in detail in our previous work [7]. It uses a modified formula to predict K_a^{app}, z and FRETmax from datasets in which the relative concentration of donor and acceptor, and the DFRET is known for a large number of cells with varied expression levels. The fitting is done via a least square model applied within an acceptor to donor ratio region of 0.2 to 3, as this region best depicts differences between different curves and does not suffer from low precision of data points (Hochreiter et al., 2019, *Sci. Rep. in press*).

$$DFRET = \frac{-\sqrt{\left(-don\, K_a^{app} - \frac{acc}{z} K_a^{app} - 1\right)^2 - 4\, don\, \frac{acc}{z}\, K_a^{app\,2}} + don\, K_a^{app} + \frac{acc}{z}\, K_a^{app} + 1}{2K_a^{app}} * \frac{FRET_{max}}{\mathbf{don}}$$

Fitting is calculated in the software R, using the non-linear least squares model, included in our own program code that we provided earlier [7].

3.8. Estimation of Distance Based on FRET$_{max}$

FRETmax is representative of the FRET efficiency E at total donor saturation, and thereby the average Efficiency of an interacting donor-acceptor pair. It can therefore be used to estimate the average distance between donor and acceptor fluorophores as based on the relation between measured transfer efficiency and distance

$$E = \frac{1}{1 + \left(\frac{r}{R_0}\right)^6}$$

Here, R_0 describes the Förster distance at which a specific pair of fluorophores produces an E of 0.5 and r describes the actual distance between donor and acceptor. However, orientation, the size of fluorophores and other factors can influence these results, which is why the results should only be seen as rough estimates and only interpreted relatively.

3.9. Prediction of Change in Free Binding Energy Due to Mutations Made at PTS1–Ligand Interface

First, Yasara Structure (Version 18.2.7) [8] was used to perform Energy Minimization experiments on two pdb structures (2c0l and 1fch) [9,10] that are complexes of the PTS1 receptor and its ligand using the AMBER15FB force field with default parameters. The energy minimized structures were then individually repaired using the RepairPDB module of the Fold × 4.0 plugin [11] in Yasara. Following this, the AnalyzeComplex (default settings, number of runs = 5) module of the FoldX plugin was used to predict the change in binding free energy (ddG_{bind}) of the complex upon mutation of the ligand at the PTS1 receptor–ligand interface.

The above method performed best in a benchmarking study we performed with mutants from Gatto et al. [1]. For each mutant, a predicted median ddG_{bind} was obtained and then correlated with the experimental ddG_{bind} values, with the pepide YQSKL as the reference ligand for each simulation. For 2c0l, simulations were conducted with its ligand truncated to a pentapeptide and separately, with the full length of the ligand retained. Correlation plots were obtained, and the Pearson's correlation coefficient and its corresponding p-value were calculated for each simulation (see Supplementary Figure 11).

After benchmarking, the above was applied to mutations made in this study using 2c0l as the model structure with ACOX3 wild type as the reference ligand. To allow for direct correlation of predicted median $\Delta\Delta G_{bind}$ with the results of this study, an experimental score was derived using the following formula:

$$\text{Score} = \log_{10}\left(\frac{220}{\text{Variant } K_{app}}\right), \text{where ACOX3 } K_{app} = 220 \text{ a.u}$$

Finally, a correlation plot was obtained and two ACOX3 variants of interest were selected for structure-based interpretation of affinity changes at the PTS1–ligand interface according to predicted models generated by the FoldX plugin. All illustrations of the PTS1–ligand interface were created using Yasara Structure.

References.

1. Gatto, G.J. Jr.; Maynard, E.L.; Guerrerio, A.L.; Geisbrecht, B. V.; Gould, S. J.; Berg, J. M. Correlating structure and affinity for PEX5:PTS1 complexes. *Biochemistry* **2003**, *42*, 1660–1666.
2. Kunze, M.; Neuberger, G.; Maurer-Stroh, S. Ma. J.; Eck. T.; Braverman, N.; Schmid, J. A.; Eisenhaber, F.; Berger, J.; Structural requirements for interaction of peroxisomal targeting signal 2 and its receptor PEX7. *J. Biol. Chem*. **2011**, *286*, 45048–45062.
3. Lametschwandtner, G.; Brocard, C.; Fransen, M.; Van Veldhoven, P.; Berger, J.; Hartig, A.; The difference in recognition of terminal tripeptides as peroxisomal targeting signal 1 between yeast and human is due to different affinities of their receptor Pex5p to the cognate signal and to residues adjacent to it. *J. Biol. Chem.* **1998**, *273*, 33635–33643.
4. Rizzo, M.A.; Springer, G.H.; Granada, B.; Piston, D.W.; An improved cyan fluorescent protein variant useful for FRET. *Nat. Biotechnol.* **2004**. *22*, 445–449.
5. Kunze, M.; Malkani, N.; Maurer-Stroh S, Wiesinger C, Schmid JA, Berger J Mechanistic insights into PTS2-mediated peroxisomal protein import: The co-receptor PEX5L drastically increases the interaction strength between the cargo protein and the receptor PEX7. *J. Biol. Chem.***2015**, *290*, 4928–4940.
6. Baes, M.; Gressens, P.; Baumgart, E.; Carmeliet, P.; Casteels, M.; Fransen, M.; Evrard, P.; Fahimi, D.; Declercq, P. E.; Collen, D., van Veldhoven, P. P.; Mannaerts, G. P. A mouse model for Zellweger syndrome. *Nat. Genet.* **1997**, *17*, 49–57.
7. Hochreiter, B.; Kunze, M.; Moser, B.; Schmid, J. A. Advanced FRET normalization allows quantitative analysis of protein interactions including stoichiometries and relative affinities in living cells. *Sci. Rep.* **2019**, *9*, 8233.
8. Krieger, E.; Vriend, G.; YASARA. View—molecular graphics for all devices—from smartphones to workstations. *Bioinformatics* **2014**, *30*, 2981–2982.
9. Stanley, W. A.; Filipp, F. V.; Kursula, P.; Schuller, N.; Erdmann, R.; Schliebs W, Sattler M, Wilmanns M Recognition of a functional peroxisome type 1 target by the dynamic import receptor pex5p. *Mol. Cell.* **2006**, *24*, 653–663.
10. Gatto, Jr. G. J.; Geisbrecht, B. V.; Gould, S. J.; Berg, J. M. Peroxisomal targeting signal-1 recognition by the TPR domains of human PEX5. *Nat. Struct. Biol.* **2000**, *7*, 1091.
11. Schymkowitz, J.; Borg, J.; Stricher, F.; Nys, R.; Rousseau, F.; Serrano, L. The FoldX web server: An online force field. Nucleic. *Acids. Res.* **2005**, 33, W382–W388.
